# Status report on novel intraoral scanner-based registration method of axes of rotation of the mandible: Proof of potential applicability and technological glass ceiling of in-house development

**DOI:** 10.1371/journal.pone.0342893

**Published:** 2026-02-23

**Authors:** Laura Lengyel, Balazs Laczi, Antal Nagy, Emil Segatto, Gergo Balazs, Arpad Safrany-Fark

**Affiliations:** 1 Department of Oral and Maxillofacial Surgery, Albert Szent-Györgyi Medical School, University of Szeged, Szeged, Hungary; 2 Department of Image Processing and Computer Graphics, Faculty of Science and Informatics, Institute of Informatics, University of Szeged, Szeged, Hungary; 3 Independent Researcher (Dentist), Budapest, Hungary; Justus Liebig University Giessen, GERMANY

## Abstract

A fast, equipment- and radiation-free input methodology for digital articulation is desirable for basic dental and surgical procedures of relatively low complexity. Our team previously proposed an intraoral scanner (IOS)-based strategy for calculating the mandibular axis of rotation using dual bite registrations in open and closed positions. In this study, we aimed to develop a methodology for validating this registration system using a state-of-the-art jaw motion tracking device (Modjaw). The bite alignment accuracy of the scanner used (*Medit i600*) proved sufficient for the task; however, its technical settings allow processing of only a limited number of bite positions, thereby restricting the number of cases that can be analysed. While in-house, insoluble technical limitations restricted the number of participants to four, significantly limiting the generalizability of the results, several main trends nevertheless appear to emerge: We verified our previous results regarding the error-induced effect ratios (EcD and AEcFE) and provided strong in-vivo support for our earlier in-vitro findings. Notably, alignment of axes obtained with our IOS-based method closely resembled the “*parallel error direction*” described previously. The reproducibility of our methodology approaches the desired 0.1 mm error threshold, but when compared with our control tool (Modjaw), it does not yet meet this criterion. The level of inaccuracy suggests that the goal remains achievable; with protocol modifications, the method could become an acceptable alternative for virtual articulation. Our findings highlight persistent clinical dilemmas and underline that many patient safety concerns remain unresolved, even in the era of advanced digital technologies. We proposed a renewed digital version of the conventional analogue control setup of articulation methods by dynamic motion registers, as a potential strategy to establish and maintain patient safety standards. Although integrating different digital platforms poses significant technological challenges, automated metrics and comparative setups offer advanced possibilities compared to the analogue experimental era.

## Introduction

**Fig 1:**
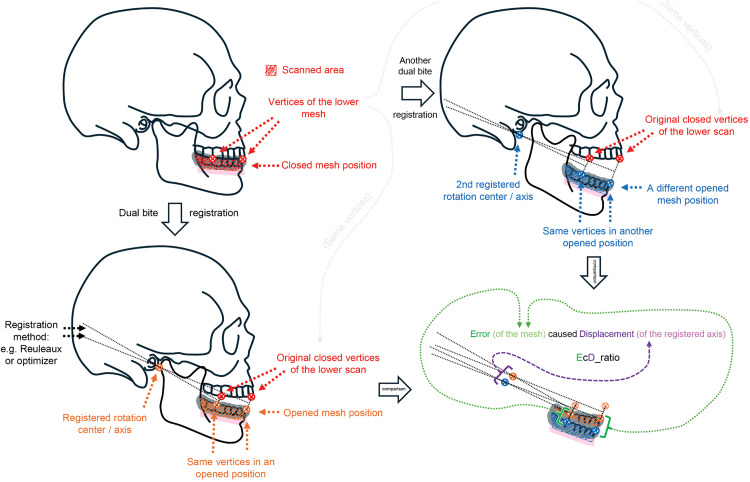
Deductive illustration of the EcD_ratio. The schematic demonstrates how the *dual-bite–based* registration is affected when one of the meshes (in this example, the opened-position scan) is displaced. Such displacement alters the resulting registered axis, as shown.

A fast, equipment- and radiation-free input methodology for digital articulation would be necessary for basic dental and surgical procedures of relatively low complexity. Our team proposed an intraoral scanner (IOS)-based strategy to calculate the mandibular axis of rotation using dual bite registrations in open and closed positions [1]. Our further investigations highlighted a core issue in instantaneous center of rotation (ICR)-based registrations and led us to similar conclusions as Mehl’s work [2,3]: even a minimal registration error and/or translational component can shift the calculated ICR to an apparently ‘*illogical*’ and clinically unacceptable extent. However, if we accept that this phenomenon follows a strict and highly predictable logic, we can begin to establish a robust framework to evaluate registration data based on evidence-driven patient safety standards. Recently, we examined two systems from the perspective of error-consequence logic, focusing on error-induced effect ratios. The first system evaluated the impact of translational errors on the displacement of the calculated axis of rotation – based on our own registration protocol. The key metric representing the internal logic of this system was the EcD_ratio: *Error* (- of the scan) *caused Displacement* (- of the axis – aka ‘d_axis_’ – based on similar values proposed by Mehl [2]), see Fig 1.

**Fig 2:**
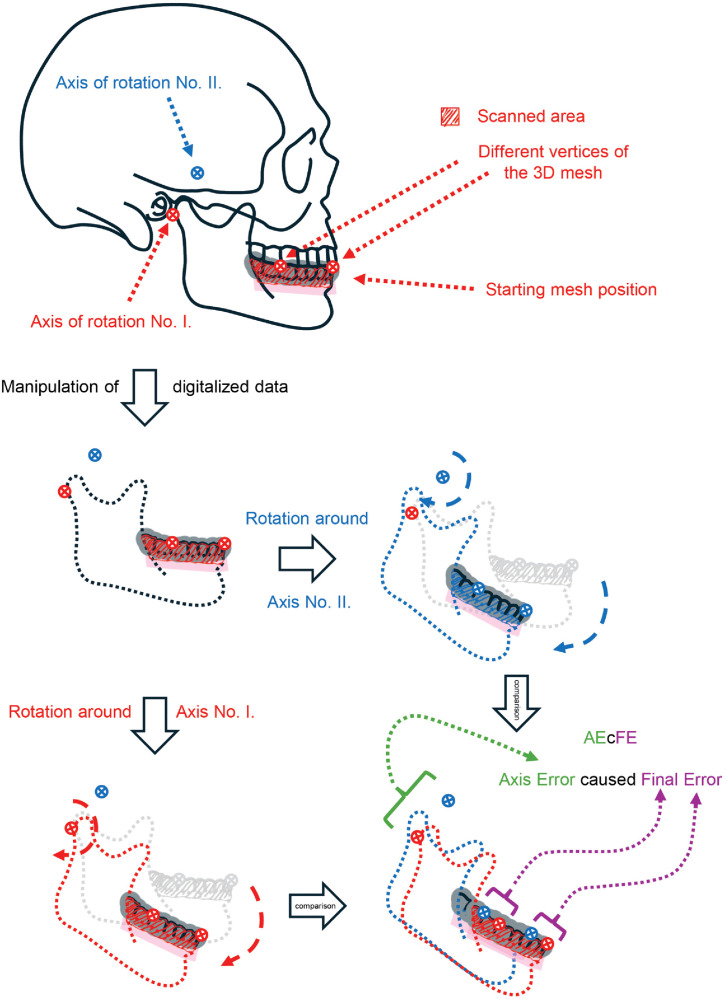
Deductive illustration of the AEcFE. The figure shows two different axes and the resulting misalignment of the same object when rotated around each axis. The positional difference of the axes leads to the observed discrepancy in the final location of the mesh.

The second system simulated inaccurate articulation of virtual casts and axes. In this setup, we performed rotations around both a “correct” and an “incorrect” axis, similar to how virtual articulators function, and measured the impact of axis error on the final mesh position. The corresponding metric was AEcFE: *Axis Error caused Final Error* (- of the mesh position after rotation), see Fig 2. All experiments were conducted digitally in an in vitro-like environment, using predefined parameters to reveal the underlying logic of the two systems.

Based on the insights gained from these experiments, we now aim to develop a methodology for validating our IOS-based registration system using a state-of-the-art jaw motion tracking device (Modjaw – Villeurbanne, France) as a reference tool. We performed repeated axis of rotation registrations with both systems and tested these axes within the virtual articulator (focusing only on rotation) simulation setup of our previous study [3]. For clinical validation, we adopted the commonly used threshold of 0.1 mm as the benchmark for acceptable accuracy. Some authors, however, argue for tolerances below 100 µm when physiological or prosthetic factors are taken into account, although IOS’s bite alignment accuracy rarely reaches such precision levels [4–7].

The origins of our dual-bite registration protocol lie in an effort to create a framework for orthognathic surgery planning based on open-source software, building on the methods of Stamm et al. [8]. Our experience gained through open-source–based development proved essential for recognizing the practical limitations encountered after a forced transition to commercial software, and for identifying the lack of a coherent conceptual foundation in many alternative approaches. Not only the rotation methods, but several other features rely on cumbersome or suboptimal solutions that are not feasible for average users without advanced modelling skills. At the same time, possession of this skill set offers substantial opportunities to explore both the theoretical and practical aspects of the system. One of the greatest challenges is the integration of multiple digital platforms, which is demanding yet essential for creating adequately controlled experimental setups. This is particularly true for largely closed systems that are mutually incompatible but nevertheless must be made to function together. Overcoming these obstacles requires an interdisciplinary team of computer scientists, 3D modellers/CAD technicians, and clinicians with strong communication and effective knowledge transfer. Nevertheless, despite all efforts, a technological glass ceiling of in-house development may ultimately be reached.

Thus, we must develop an appropriate control strategy not only for the methods used in surgical planning, but also for virtual articulator systems in general, if we are to avoid continuing to neglect fundamental patient safety obligations. The spatial linking of digital information originating from different systems – *sometimes even within the same system* (e.g., bite alignment accuracy) – represents the most critical point of vulnerability, as it connects scanned objects or anatomical surfaces, segmented bone structures, intraoral scans, and digital counterparts of analogue elements (e.g., face-bow or scan body systems), and is often taken for granted by both authors and developers. Based on our previous experience, derived from purely theoretical basic research, even the smallest inaccuracy can be amplified into a substantial error, while in other cases the system may exhibit remarkable error tolerance [3]. Virtual articulation is therefore not a straightforward or inherently reliable tool, but rather an exceptionally complex problem space that demands appropriate scientific humility and rigorous research effort.

## Materials and methods

**Fig 3:**
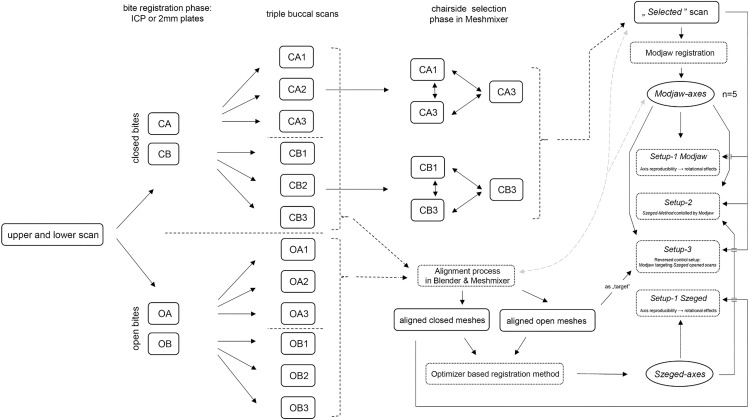
Schematic representation of the clinical and digital workflow, their outputs, and the relation to the different experimental setups.

Four dentists, acting as ‘*patients*’, were recruited to develop a clinical protocol suitable for a Modjaw-controlled study of our IOS-based registration method (due to the simpler designation of the protocol we called it ‘*Szeged-method’* after the hometown of the team; all axes produced by our IOS-based protocol will be referred as *‘Szeged-axes’*, as well as axes registered with Modjaw will be titled as *‘Modjaw-axes’*). This study was approved by the Scientific and Research Ethical Committee of Medical Research Council (ETT TUKEB) of the Hungarian Ministry of Interior (approval no.: BM/13110–3/2025). Written consent was obtained from the participants; the recruitment period lasted from 15.07.2025–24.08.2025. Upper and lower jaw scans were performed, and special composite bite blocks were fabricated for all four patients by the same clinician (for an overview of the workflow, see S1 Presentation and Fig 3.). These bite blocks were produced in a similar manner to the previously described method for the open-mouth position [1]. The only difference was that only two pairs of bite blocks were prepared, each containing approximately 2 mm thick composite plates (the protocol was simplified by omitting the 1 mm and 3 mm plate versions).

For each participant, two closed and two open bite positions were registered (closed: CA & CB; opened: OA & OB). As in the previous protocol, each bite position was scanned three consecutive times without removing the bite block or opening, to assess the alignment accuracy of the IOS (marked with numbers: 1,2,3, thus case names contained 3 characters, e.g., CA1 or OB3 etc.). The main difference in the current protocol was that the ‘closed bite position’ was defined differently, in order to adapt the clinical workflow to the control tool, the Modjaw. In the previous protocol, the closed bite was captured at the first point of occlusal contact, assuming that a centric relation (CR) position of the temporomandibular joint (TMJ) had been achieved through gentle guidance of the mandible. However, the fundamental methodological differences between the two registration processes made the development of a unified clinical protocol challenging. While the motion-tracking process collects data during the free movement of the mandible, the dual bite-scan method requires a highly stabilized, fixed position of the dental arches during registration. The typical starting point for Modjaw registration is habitual occlusion, or intercuspal position (ICP). However, some authors propose more complex protocols to achieve a more favourable condylar position [9,10]. In certain clinical situations (*e.g., full-arch restorations, “maxilla-first” osteotomies, cases where ICP is lost, or when TMJ repositioning is the primary goal*), these protocols become central to the therapy. Nonetheless, our aim was to develop a simple, immediate tool suitable for everyday clinical use in straightforward cases. Therefore, we kept all aspects of the developed process as simple and efficient as possible. At this stage, we decided to use the ICP as the “*closed bite position*” for the dual-bite and IOS-based registration.

For Modjaw registration, one scan was selected from six *closed* bite scans that had been acquired during the IOS-based registration protocol which involved scanning the patient three times consecutively in a closed position without opening, followed by a brief relaxed opening. A duplicated case file was then created, and after the patient returned to closure, the triple scan was repeated. This resulted in two groups of three scans each. The selection for Modjaw was performed chairside immediately using Meshmixer. All six scans were loaded into the software, and the *deviation tool* of the software was used to compare each scan within its respective group (*i.e., intra-group comparison only*). Differences below 0.01 were disregarded as clinically negligible (*one-tenth of the clinical error threshold*). Groups with deviation values approaching 0.1 were not preferred. To streamline data handling, CB1 was selected in the first three cases. However, in Case IV (the last chronologically), CB1 exhibited nearly unacceptable deviation (~0.1), compromising our aim of consistently selecting CB1; therefore, CA2 was used instead (S1 Table). The chosen scan (one of each participant) was used for Modjaw registration and labelled as “*selected*” for further investigations (see later).

The Modjaw registration protocol involved consecutive opening and closing movements performed by the patients, characterized by limited opening range (~5–10 mm) and multiple fast cycles (n = 20+), see S1 Video. Historically, the ‘*true hinge axis*’ theory was applied to the initial phase of jaw opening. Regardless of ongoing academic debate over its existence (*the authors' opinion on this has been discussed previously* [1,3]), it remains a logical strategy to calculate the rotational axis from the early stages of opening, before the onset of translational phase of the TMJ movement (*In this article, we use classical gnathological terminology. Although recent and well-respected critiques of the classical nomenclature exist, we have chosen to retain this terminology for the sake of clarity and consistency until a consensus on the revised terms is established*). The full range of the registration of all repeated opening and closing cycles was used for the axis calculation. Five such registration sequences were recorded consecutively, separated only by short resting periods.

### Technical difficulties

During our previous preliminary investigations, we identified two main technical obstacles in our IOS-based protocol: (1) bite alignment inaccuracy of the scanner, and (2) the positioning and post-processing protocol of the IOS, which resulted in misaligned exported STL files. These misalignments rendered the data unsuitable for axis registration, as our method requires identical meshes perfectly aligned through the upper arches. In contrast, the scanner produced inconsistently displaced meshes with altered surfaces, due to the unavoidable post-processing steps required during scanning. For this control study, we used a different IOS with improved performance and more suitable settings: the *Medit i600* (Medit, Seoul, South Korea). This scanner allows scanning up to five different bite positions within a single case. Consequently, after post-processing, all five STL sets were perfectly aligned and identical. Moreover, bite alignment accuracy was superior compared to the previous scanner. (This improvement – likely due to the time elapsed since the last trial and the rapid evolution of IOS technology – was the minimal development we had predicted in our earlier publication and the prerequisite we awaited to continue our clinical trials [1].)

Our initial impression of the selected tool was positive, as the built-in multi-bite scan feature seemed suitable for case acquisition. However, the scanner has a limitation in the number of bites that can be scanned, and it became evident that post-processing could not be skipped even if upper and lower scans were cloned before bite registrations. Consequently, the exported STLs were no longer identical and were positioned differently. These properties make the selected IOS suitable only for single-case registration. Large-scale studies involving multiple bite positions and continuous scans exceed its capabilities. Therefore, we had to perform a modified version of the CLVs method we previously described [1]. This is a manual 3D modeling process used to realign mesh sets based on the upper arches and to substitute the differently processed STLs with identical ones. The modifications were necessary because the CLVs were less stable in their 3D positions with the *Medit*, resulting in inaccurate realignments. To aid this process, we used the ‘*Align to Target*’ tool in Meshmixer (Autodesk, San Francisco, CA, USA) for an initial alignment step. Since all meshes were derived from the same raw dataset, this method worked reliably. Nevertheless, the scientific team – accustomed to high-precision 3D modelling – regarded this step as a frustrating necessity, as it introduces a theoretical risk of error. Close supervision of the automated process is essential; the ‘*Deviation*’ tool in Meshmixer was found to be appropriate for this purpose.

A modeling step was also required to derive the coordinates of the Modjaw axes. Cylinder objects representing the calculated axes can be exported from the software, with the center point at each end of the cylinder defining the linear axis. To extract these coordinates, the vertices of the circle at one end were selected, and a free vertex was moved to the ‘*center of the selection*’ using shortcuts in Blender v2.79 (Shift+S: ‘*cursor to selected*’; then, after selecting the free vertex, ‘*selection to cursor*’). The same procedure was repeated at the other end of the cylinder. (The coordinates can be found under “N” menu.) This represents one possible approach, although several alternative methods could be applied to achieve the same result.

**Reproducibility warning:** Meshmixer alters the vertex order of meshes. Therefore, if vertex-level labelling is needed for subsequent registration, unmanipulated meshes must also be imported and exported accordingly. While the IOS proved technically capable for registration, its limitations remain a significant barrier. Even for a limited number of participants, the required manual 3D work was immense. This makes large-scale studies involving multiple clinical protocols practically impossible. Ironically, the IOS appears technically suitable for clinical use, but is hindered only by the lack of clinical evidence. These findings suggest that further in-house development has reached a technological glass ceiling without corporate collaboration. Another unfortunate consequence is that these stringent technical requirements significantly restrict the testing of multiple scanners from different manufacturers. However, if any interested author finds an IOS that meets these criteria, all necessary materials for reproducing our investigation are available in the Data Repository of the current and previous papers [1,3].

**Methodological Dilemmas:** 1) Defining axes using pairs of coordinates (*x, y, z*) poses a significant challenge. At which point along the defined line should the axis be evaluated or validated? In our protocol, we typically defined the start and end points of the axis as being 200 mm apart, measured 100−100 mm from the point on the axis closest to the center of gravity of the given mesh. However, when manipulating multiple meshes, different coordinate sets can occasionally be generated for the same axis. This is an unfortunate but unavoidable consequence of the experimental setup. While this variation typically introduces only minor discrepancies that do not affect the overall conclusions, it remains a technical limitation.

2) The second dilemma arises when evaluating mesh-to-mesh distances. We typically used the root mean square (rms) error of all corresponding vertex pairs. This method proved appropriate for calculating derived parameters such as the EcD_ratio or AEcFE. However, it only loosely reflects clinical scenarios, since dental arches do not penetrate one another – in reality, the first point of contact halts or redirects further movement. To address this limitation, we performed double measurements: in addition to the rms_error, we also calculated the maximum distance between all vertex pairs. This secondary metric may prove to be a more clinically relevant indicator in future analyses.

Future note

The presented manual modeling workflow used to overcome alignment- and mesh surface-related issues could, in principle, be automated using optimizer approaches similar to our two-stage axis registration method [1]. The positional changes introduced by multiple translation, rotation, and mesh substitution steps can be adjusted to the specific output characteristics of the scanner’s built-in software and the given experimental setup. However, such automation would require continuous fine-tuning and maintenance.

In routine clinical use with the Medit system, the manual workflow is not required, as the limited number of bite registrations needed per case is already supported by the software. The constraints become relevant primarily in multi-case validation research, where the number of required registrations exceeds the software limits. A major obstacle is that each scanner brand – and in some cases even different models – would require a distinct automation algorithm, which would likely remain functional only until a subsequent software update, where even minor changes in output properties could unintentionally compromise the entire process. Scanner-specific output handling and frequent software updates therefore introduce variability that is difficult to manage without direct corporate assistance.

Notably, this issue could be resolved by the Manufacturers with only minor modifications to the software output, such as aligning exported STL files by the upper arch (defined by mass center and local orientation) and optionally applying post-processing prior to the bite scan, which would effectively replace the need for the current manual workflow.

### Registrations

For the IOS-based method, we applied our previously published optimizer-based Iterative Closest Point (ICP) algorithm, utilizing a simplified Euclidean distance metric and the Levenberg-Marquardt optimizer (Shown in Fig 3. of our previous study [1] available at https://doi.org/10.1371/journal.pone.0285162.g003). The open meshes were rotated, while the corresponding closed ‘*fixed*’ meshes served as the targets of the transformation. The resulting registered axes were named according to the two meshes involved (e.g., CA1:OA1).

The EcD_ratio was calculated by assigning a single theoretical ‘*fix’* mesh as the target of two other (e.g., the same closed case CA1 – *fix* – served as the target for both OA2 and OB3 opened scans, the positional difference between the resulting axes – d_axis_ of *CA1:OA2* and *CA1:OB3* axes – was caused by the misalignment of the two opened meshes OA2 and OB3 – quantified as rms_error or max_error; for the purpose of EcD value calculation, open scans were also temporarily designated as theoretical *fixed* meshes.).

All registered axes were tested through rotational simulations (compared with each other or with the control axes – Modjaw in *Setup-2*), mimicking their use as primary inputs in a digital articulator. For each participant in *Setup-1 Modjaw* and *Setup-2* the previously ‘*selected*’ mesh was placed in our virtual articulator and subjected to rotations of 1°, 2°, and 3° (approximately equivalent to 1.3 mm, 2.6 mm, and 3.8 mm of mouth opening) in both positive and negative directions. In *Setup-1 Szeged* all *closed* meshes were used. These rotations simulated mandibular autorotation following various clinical interventions, such as surgical maxillary impaction, molar intrusion, or bite elevation etc.

The base unit of the experimental setup was presented in Fig 3. of our previous study [3] available at https://doi.org/10.1371/journal.pone.0329021.g003; with the only difference in the names of the axes of the setup: the registered *Szeged-* and *Modjaw-axes* acted as ‘*central*’ and ‘*modified*’ axes with different combinations determined by the given *Setup*).

Intra-group comparisons were conducted to evaluate the reproducibility of our registration method and its clinical reliability, with a focus on the 0.1 mm error threshold. The same reproducibility assessments were also performed on the Modjaw-derived axes. These were labeled as *Setup-1 Modjaw* and *Setup-1 Szeged*.

In addition, we compared the performance of the axes derived by the *Szeged-method* with those produced by Modjaw, designated as *Setup-2*.

A further experiment (*Setup-3*) was conducted in which the ‘*selected’* closed meshes were rotated using the Modjaw-derived axes until optimal overlap with the open meshes from the IOS-based acquisition was achieved, defined by the lowest Euclidean distance metric. This procedure was repeated for each open scan.

For all setups, the following metrics were recorded: d_axis, rms_error, maximum_error, angular deviation between compared axes, and derived parameters such as the AEcFE ratio.

## Results

### IOS bite alignment accuracy, EcD_ratio and d_axis_ values

All bite positions were scanned three times in quick succession, without any opening movement. Pairwise distance measurements were performed between the three meshes of each bite position (e.g., CA1–CA2, CA2–CA3, and CA1–CA3) using Meshmixer’s *Deviation* tool to evaluate the maximum distance within each mesh group. The average maximum distance across all four participants was 0.036 ± 0.030 mm in the closed-bite groups, with only one case (Case III: CA2–CA3) exceeding the 0.1 mm clinical limit (S1 Table). The open-bite group showed lower accuracy, with a 0.145 ± 0.066 mm mean maximum deviation, and approximately 75% of cases exceeding the clinical limit. Since IOS performance is likely not optimized for scanning in an open-bite position, the reduced accuracy may be due to untested clinical scenarios from the perspective of the alignment algorithm, or the less stable mandibular position compared to ICP.

For pairwise distance measurements carried out by the automated algorithm (*not via Meshmixer*) for all closed (CA & CB groups combined) and open cases (OA & OB groups combined), see S2 Table – “*rms & max_error values*” Sheet Tab. These results show that introducing an opening between the two sets of bite scans, as well as remanufacturing the bite block, slightly increased the measured values. Caution is advised when comparing datasets, as the Meshmixer and automated algorithm measurements are not identical.

The EcD_ratio values indicate how tolerant the *Szeged-method* registration was to positional error. Here, error is defined as “*error from the perspective of pure rotation*” with any physiological translational components or technological positioning inaccuracies affecting the final calculated axis of rotation. If such components are present during registration, the resulting axis should technically be defined as the *instantaneous center of rotation* (ICR), since the motion was not purely rotational but simplified as such (see [1,3] for a detailed discussion). The EcD_ratio can be interpreted as the number of millimeters by which a registered axis would shift in response to 1 mm of registration error (either rms or maximum values can be used for calculation). S2 Table – “*EcD_ratio_Szeged_Method*” Sheet Tab – lists all EcD_ratio values. The observed ratio 36.69 ± 22.83 (: 1) closely matches our previous clinical registration results (41.83 ± 14.89). Comparisons between cases with the same clinical bite position showed greater error tolerance than “mixed bite” comparisons. (‘same bite’ vs ‘mix bite’ in S2 Table).

Preliminary evaluation of the registered axes revealed no major errors in the IOS-based registrations that would indicate clinical or technological acquisition problems (in our previous study, cases with significant registration errors were excluded). However, two Modjaw registrations produced exceptionally high d_axis_ values (highlighted in red and crossed in S2 Table – “*d_axis Modjaw*” Sheet Tab). After excluding these two cases, the average d_axis_ value was 1.71 ± 0.59 mm (Case I: 1.62 ± 0.61 mm; Case II: 1.72 ± 0.66 mm; Case III: 2.03 ± 0.66 mm; Case IV: 1.56 ± 0.47 mm), compared to 2.78 ± 1.81 mm for Participant I and 4.32 ± 3.02 mm for Participant III without exclusion. These two axes were excluded, as they were considered the result of inadequate registration. For completeness and transparency, all further calculations were also performed with and without these exclusions wherever possible, as shown in the corresponding Supplementary Tables; however, our main conclusions in this paper focus on the datasets with exclusions applied.

### Results of *Setup-1* Szeged and *Setup-1* Modjaw

Many authors of the last century set the clinical goal of articulation such that the registered axis should be within 3–5 mm (*in some cases up to 6 mm*) of the ‘*true axis*’ – usually determined by axiography – resulting in less than 0.1 mm clinical error at the level of the occlusal surfaces [4,11,12] – or at least claimed/ expected to be less (*later discussed*). Conventional analogue, facebow-based articulation methods meet these criteria in 20–98% of cases (Walker: 20%; Piehslinger: 23%; Lauritzen and Bodner: 33%; Teteruck and Lundeen: 56.4%; Beyron: 87%; Schallhorn: 98%) according to these authors [4,13–17]. The IOS-based method’s reproducibility meets ‘*similar*’ (*The digital variant of these investigations, which could be interpreted as applying the ‘same’ rather than merely ‘similar’ criteria, can be found under Setup-2 – practically the digital version of the analogue facebow- and axiography- based setups.*) criteria in approximately 65.1% of cases, if maximum error – rather than the rms_error value – is considered clinically relevant (Table 1). However, the mesh surface sites producing the maximum error may be located at the extremities of the scan, e.g., on the gingival parts. This makes the criterion somewhat too strict, whereas rms_error would be too lenient.

**Table 1:** The registered parameters of *Setup-1 Szeged.*

(mm/ °)		rms_error	max_error	d_axis__AVG start&end	angular deviation of axes
**ALL**	AVG	0.057	0.094	5.391	1.28
	SD	0.046	0.070	3.092	0.83
(n)	>0.1 mm	675	1509		
(n)	<0.1 mm	3645	2811		
(%)	<0.1 mm	84.4	65.1		
**CASE I**	AVG	0.028	0.053	3.352	0.83
	SD	0.016	0.032	1.463	0.44
(n)	>0.1 mm	0	108		
(n)	<0.1 mm	1080	972		
(%)	<0.1 mm	100.0	90.0		
**CASE II**	AVG	0.085	0.111	5.198	1.97
	SD	0.066	0.080	2.550	1.01
(n)	>0.1 mm	432	433		
(n)	<0.1 mm	648	647		
(%)	<0.1 mm	60.0	59.9		
**CASE III**	AVG	0.048	0.098	5.956	1.30
	SD	0.026	0.059	2.528	0.78
(n)	>0.1 mm	47	464		
(n)	<0.1 mm	1033	616		
(%)	<0.1 mm	95.6	57.0		
**CASE IV**	AVG	0.064	0.116	7.060	1.04
	SD	0.041	0.078	3.992	0.47
(n)	>0.1 mm	196	504		
(n)	<0.1 mm	884	576		
(%)	<0.1 mm	81.9	53.3		

Note: these d_axis_ values are analogue with S2 Table - “*EcD_ratio_Szeged_method” –* Sheet Tab - “C fix” values.

Reproducibility issues increased with greater amounts of opening. While 1° of opening produced maximum error values below 0.1 mm in 97% of cases, this proportion decreased gradually with greater rotation, ~ 62% in the 2° group and ~34–37% in the 3° group (Table 2). This can be explained by the different AEcFE values of these groups: the 1° group tolerated positional differences of the axes well (~120 mm distance between axes would produce 1 mm maximum error at the scan level), whereas this ratio decreased to ~60:1 and then to ~40:1 with increased rotation angles. The participants produced similar mean AEcFE values (Table 3), indicating that the level of error tolerance was relatively constant.

**Table 2:** The different mean error and AEcFE values of all four participants divided by the amounts of rotation.

error (mm):	rms	max	rms	max	rms	max
	**+1° & −1°**		**+2° & −2°**		**+3° & −3°**	
AVG	0.028	0.047	0.057	0.094	0.085	0.142
SD	0.019	0.027	0.037	0.054	0.056	0.081
	**+1°**		**+2°**		**+3°**	
AVG	0.028	0.047	0.056	0.095	0.084	0.143
SD	0.018	0.027	0.037	0.054	0.055	0.081
>0.1 mm (n)	0	24	126	274	202	475
<0.1 mm (n)	720	696	594	446	518	245
<0.1 mm (%)	100.00	96.67	82.50	61.94	71.94	34.03
	**−1°**		**−2°**		**−3°**	
AVG	0.028	0.047	0.057	0.094	0.086	0.141
SD	0.019	0.027	0.038	0.054	0.057	0.081
>0.1 mm (n)	0	22	135	264	212	450
<0.1 mm (n)	720	698	585	456	508	270
<0.1 mm (%)	100.00	96.94	81.25	63.33	70.56	37.50
**AEcFE ratio** (rms & maximum values used):						
	**+1° & −1°**		**+2° & −2°**		**+3° & −3°**	
AVG	209.25	116.87	104.51	58.42	69.55	38.93
SD	69.76	61.41	34.83	11.59	23.17	7.73

**Table 3:** The different AEcFE values of the four participants.

AEcFE ratio (values used:)		rms_error	max_error
**ALL**	AVG	127.8	71.4
	SD	75.7	36.6
**CASE I**	AVG	141.5	77.8
	SD	76.6	39.4
**CASE II**	AVG	91.1	62.2
	SD	60.2	32.3
**CASE III**	AVG	147.2	74.2
	SD	78.2	36.9
**CASE IV**	AVG	131.3	71.4
	SD	73.5	35.6

The Modjaw’s reproducibility was, as expected, far superior. The majority of cases met the 0.1 mm maximum error criterion in our virtual articulator model, and the axes were distributed within a ~ 2 mm radius (Table 4). We therefore accepted our Modjaw protocol as a sufficient clinical control tool for rotation axis registration. It is important to note that a perfect registration tool is unlikely to exist, as minimal translational components or other ‘*errors*’ will probably compromise the stability of axis position acquired by pathographic methods, as noted by Mehl [2]. All other relevant information on *Setup-1 Modjaw* is available in Table 4 and S4 Table.

**Table 4:** The registered parameters of *Setup-1 Modjaw.*

(mm/ °)		rms_error	max_error	d_axis__AVG start&end	angular deviation of axes
**ALL**	AVG	0.035	0.045	1.707	0.50
	SD	0.021	0.025	0.568	0.29
(n)	>0.1 mm	4	16		
(n)	<0.1 mm	380	368		
(%)	<0.1 mm	99.0	95.8		
**CASE I**	AVG	0.041	0.049	1.475	0.39
	SD	0.026	0.029	0.408	0.19
(n)	>0.1 mm	4	8		
(n)	<0.1 mm	68	64		
(%)	<0.1 mm	94.4	88.9		
**CASE II**	AVG	0.026	0.037	1.725	0.33
	SD	0.014	0.020	0.643	0.13
(n)	>0.1 mm	0	0		
(n)	<0.1 mm	120	120		
(%)	<0.1 mm	100.0	100.0		
**CASE III**	AVG	0.036	0.048	2.106	0.92
	SD	0.021	0.026	0.555	0.24
(n)	>0.1 mm	0	4		
(n)	<0.1 mm	72	68		
(%)	<0.1 mm	100.0	94.4		
**CASE IV**	AVG	0.040	0.049	1.590	0.49
	SD	0.021	0.026	0.445	0.23
(n)	>0.1 mm	0	4		
(n)	<0.1 mm	120	116		
(%)	<0.1 mm	100.0	96.7		

**Fig 4:**
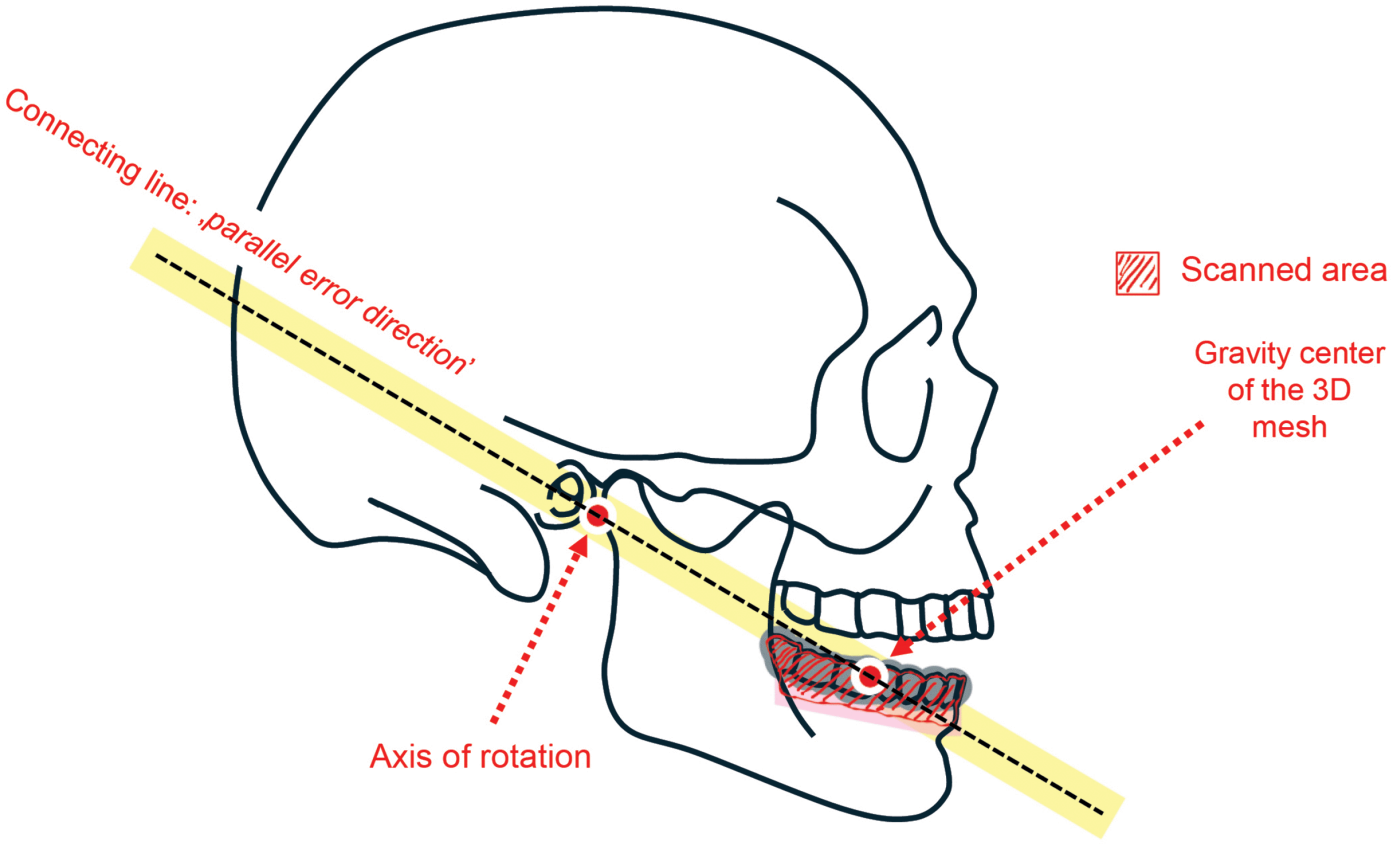
Orientation of the parallel error direction relative to the skull and the registered digital data.

Visual inspection of the axes of each participant (IOS based and Modjaw) reveals many details on the core logic of the system acting behind the surface. For 3D display.obj files are available in the data repository (.obj files are oriented “*Forward: -Z & Up: Y*” manner, some software import settings may alter this. For example, Blender should be set to ‘*Y Forward*’ and ‘*Z Up*’ import option). The coordinates of the registered ICRs can also be found in the respective supporting tables. S2–S5 Video briefly presents each case: black axes are from IOS-based registrations, while coloured axes were acquired by Modjaw. (the position of the excluded Modjaw axes – Case I: Modjaw-3 and Case II: Modjaw-2 – support our decision as they are clearly located further from the main distribution site of the Modjaw axes). The most important visual finding is that the *Szeged-axes* appear aligned with the previously described “*parallel error direction*”, see Fig 4 (The different directions are shown in Fig 2. of our previous study [3] available at https://doi.org/10.1371/journal.pone.0329021.g002). This was not the result of investigator intervention; the same optimizer algorithm was used as before, without fine-tuning. The only changes to the IOS-based registration experimental setup were: the improved bite alignment accuracy and a modified closed bite registration protocol. Seeing the axes distributed along a line was initially a surprising ‘*pinch me*’ moment for the authors – but it is entirely logical: the *parallel error direction* was proven to be the least error-sensitive of all directions (producing the highest AEcFE values in digital in-vitro settings [3]). It is therefore a ‘natural’ phenomenon that an optimizer algorithm minimizing error would find axes along the direction of least sensitivity. This serves as a clear example of fundamental research findings recurring in clinical settings.

### Results of *Setup-2* – Modjaw control of the *Szeged-method*

If we accept the superior reproducibility of Modjaw registration as sufficient validation for use as a standard, we can compare IOS-based axes against them to assess the clinical applicability of the novel method. At this stage, the goal is not to *prove* clinical applicability – case numbers are too low – but to establish a viable workflow for control investigations if manufacturers were to implement the necessary IOS setting modifications. Unfortunately, comparison of the two methods was less promising (Table 5 and S5 Table). The registered maximum error was approximately 2.5 times higher than the 0.1 mm clinical goal, with only ~10% of cases within the limit. This indicates a protocol issue that needs to be addressed. However, the results remain within potential reach of the clinical target. With further improvements, achieving the goal seems feasible, though this would require testing multiple clinical protocols with large case numbers – practically impossible with the available IOS settings. Moreover, enhanced bite alignment performance of the IOS in the open mandibular position could significantly influence the overall results. Just two years ago, such results were unattainable due to technological limitations. Recent advances in closed-bite alignment have shifted the protocol from a theoretical concept to one that is nearly applicable. If similar improvements could be achieved for the remaining half of the protocol, comparable progress might be possible; however, with our current knowledge, it is impossible to assess this prospect.

**Table 5:** The registered parameters of *Setup-2.*

(mm/ °)		rms_error	max_error	d_axis__AVG start&end	angular deviation of axes
**ALL**	AVG	0.201	0.248	8.994	3.60
	SD	0.100	0.124	2.429	1.38
(n)	>0.1 mm	3255	3522		
(n)	<0.1 mm	633	366		
(%)	<0.1 mm	16.3	9.4		
**CASE I**	AVG	0.195	0.223	8.820	3.42
	SD	0.086	0.097	1.418	0.63
(n)	>0.1 mm	726	792		
(n)	<0.1 mm	138	72		
(%)	<0.1 mm	16.0	8.3		
**CASE II**	AVG	0.234	0.286	9.273	4.97
	SD	0.114	0.139	2.145	1.25
(n)	>0.1 mm	904	1050		
(n)	<0.1 mm	176	30		
(%)	<0.1 mm	16.3	2.8		
**CASE III**	AVG	0.134	0.170	6.701	1.91
	SD	0.057	0.074	1.972	0.83
(n)	>0.1 mm	576	600		
(n)	<0.1 mm	288	264		
(%)	<0.1 mm	33.3	30.6		
**CASE IV**	AVG	0.226	0.291	10.689	3.72
	SD	0.095	0.124	2.179	0.48
(n)	>0.1 mm	1049	1080		
(n)	<0.1 mm	31	0		
(%)	<0.1 mm	2.9	0.0		

### Results of *Setup-3* – Reversed control setup

*Setup-3* was a highly theoretical investigation: As pre-defined centres of rotation are not available in a clinical setting, the exact location of each participant’s rotation axis is unknown; indeed, such an axis may not exist in real biological circumstances. From a clinical perspective this is irrelevant – what matters is a modelling method that can mimic a patient’s lower jaw motion acceptably. Since we cannot determine the absolute truth of our registrations, we cannot exclude the theoretical possibility that our control tool (Modjaw) has a technical flaw that consistently produces similar but biologically inaccurate outputs. Based on more than a century of analogue axiography experience, we reject this as a general concern, but adopting a contrarian, critical perspective may still yield insights. This was the case in *Setup-3*, which compelled the ‘clinical’ side of our team to reassess their firm opposition to what they initially saw as an ‘*unrealistic*’ and ‘*unnecessary*’ control setup “*that only makes us look* *unprofessional*”, as the results proved otherwise. The ‘*selected’* mesh of each participant was used for Modjaw registrations, and we performed rotations of these ‘*selected’* closed meshes around the registered Modjaw axes. The target meshes were those obtained during *open* IOS-based registrations. These rotations produced highly consistent maximum error values of about 0.5 mm on average (S6 Table, All: 0.508 ± 0.132; Case I: 0.555 ± 0.071; Case II: 0.584 ± 0.175; Case III: 0.367 ± 0.044; Case IV: 0.507 ± 0.062) – five times the clinical goal. Such results, by themselves, have limited value. However, the standard deviation values were far more revealing: they were highly stable – often around or even below the 0.1 mm threshold – contrasting with the positional error. Visual inspection of these meshes (the STLs are available through the data repository, S6–S9 Video present an example of each participants: in each video bright scan shows the Modjaw rotated case, dark the original IOS based open scan) clearly showed that the Modjaw axes rotated the scans into a more forward position (except Case II, which showed more of a lateral shift) than the position defined by our bite block protocol. This positional difference was practically the *same* in 100% of cases for each participant. It led to an important conclusion regarding our protocol: the bite block registration step in our clinical workflow caused the systematic misalignment of the IOS-based registration (the acquired mesh positions and the related values) in *Setup-2*’s control investigation. While this protocol-induced deviation seems straightforward here, the methodological situation may be more complex, raising well-known clinical dilemmas that require further discussion and resolution.

To facilitate interpretation of the extensive dataset presented above, a comprehensive summary table is provided below (Table 6). This table consolidates some of the key findings into an accessible form, supporting a clearer overview of the study’s comparative results.

**Table 6:** Overview table of Results. Simplified overview table of the results highlighting and summarizing some of the key findings.

Test	Involved registration method(s)	Main points of interest	Accuracy/Registered values/Relevant findings	Interpretation	Relevant Tables
Basic findings	*Szeged-Method*	Closed bite scans	All but one meets clinical standard	Bite scans accuracy in opened mandibular position needs improvement ^**A**^	S1 Table
		Opened bite scans	75% does not meet clinical standard		S1 Table
		EcD ratio	36.69 ± 22.83 (: 1)	The observed ratio closely resembles previous results (41.83 ± 14.89)	S2 Table
	Modjaw.	d_axis_ values	Two cases produced exceptionally high d_axis_ values	Judged as registration failure, exclusions were made	S2 Table
*Setup*-1.	*Szeged-Method*	Clinical acceptability of reproducibility	Only 1° rotation group produced acceptable results	Clinical applicability remains within reach, provided further clinical and/or technological refinement; for example, the systematically greater inaccuracies observed in the opened position of the dual-bite protocol —compared with the closed position— should be resolved, see: (**A**).	Table 1, S3 Table
	Modjaw	Clinical acceptability of reproducibility	Optimal reproducibility	Previously (‘in-vitro’) described *parallel error direction* reappeared in clinical settings	Table 4, S4 Table
	*Universal finding*	AEcFE values	AEcFE values decreased with increased rotation angles	Previously described fundamental logic of AEcFE reconfirmed	Table 2, S3 Table
*Setup*-2	Modjaw used as ‘*golden standard*’	Rotations along *Szeged-axes* compared to control rotations by *Modjaw-axes*	AVG maximum error 2.5 times higher than the 0.1 mm clinical goal.	The current error magnitude exceeds clinical standards, yet remains sufficiently close that further refinement ^**B**^ could realistically bring it within the clinically acceptable range.	Table 5, S5 Table
			Only ~10% of cases meet 0.1 mm	Main methodological difference:	
				Modjaw provides dynamic mandibular motion registration	
				*Dual-bite* registration uses two static mandibular positions	
*Setup*-3	*Selected* ‘closed’ scans rotated by *Modjaw-axes*	Where would be the lower arch during the dynamic Modjaw registration after opening movement (performed artificially by rotations along the *Modjaw-axes*) compared to the static opened position of the Szeged *dual-bite* protocol?	Highly consistent maximum error values:	Raises many questions regarding the clinical protocol.	S6 Table
			AVG 0.5 mm; SD ~ 0.1 mm.	See Discussion section	
	Target: Szeged ‘open’ scans		Modjaw axes rotated the scans into a more forward position (except Case II, which showed more of a lateral shift) than the position defined by our bite block protocol.	Also reveals the main cause of clinical protocol-induced deviation of *Setup-2*, see: (**B**)	

## Discussion

When interpreting the results, caution is warranted, as the limited number of cases precludes far-reaching conclusions. Further studies will be required, once the identified technological constraints have been resolved (ideally by the *Manufacturer*), to validate or re-evaluate the trends observed.

The *Setup-1* protocols of each registration method demonstrated a certain level of reproducibility. From a clinically oriented scientific perspective, we can approve the reproducibility of the presented Modjaw protocol as a control tool for further investigations. Our results may also serve as a basis of comparison for other research groups seeking to refine different Modjaw based workflows focusing on rotation axes. The reproducibility of our IOS-based methodology approaches the desired 0.1 mm error threshold when applying the (perhaps overly strict) *maximum error parameter*. However, when compared to our control tool – the Modjaw – it does not yet meet the criteria. The level of inaccuracy suggests that the goal is not unattainable; with protocol modifications that improve our open bite position, an acceptable alternative for virtual articulation may be achievable. While the technological background has improved markedly since our last trial and appears optimal from an instrumental perspective, the main obstacle now lies in the processing stage. With the current settings, weeks – if not months – of manual modelling is required to process even a handful of cases, making the testing of multiple clinical protocols practically impossible.

The alignment of axes obtained from our IOS-based protocol closely resembles the *parallel error direction* described previously [3]. This provides strong support for our earlier in-vitro findings and suggests that our proposed digital in-vitro laboratory of complex motions is more than a theoretical construct. These concepts have found their own clinical confirmation, providing *real-life* evidence of their relevance – much to the authors’ satisfaction.

The source of our main limitation – the open bite position – reopens long-standing debates about interocclusal registration: ICP versus complex protocols referencing specific TMJ positions. In cases such as complex occlusal rehabilitation, full dentures, or ‘*maxilla-first’* surgical protocols, options are restricted by the absence of ICP. Some authors combine deprogramming with Modjaw registrations, such practices might also be applicable for control strategies in the future [9,10]. Further, there is the old question of body posture: our bite block method works only with the patient in a horizontal chair position, as bite blocks are not fixed to the teeth. Different clinical applications may require different protocols. For example, in surgery the patient is supine under anesthesia, with a relaxed musculature and a mobile maxillary complex; in such cases, the body posture and gentle manual guidance during our open bite composite block protocol might not be the weak point. However, it revealed several points of methodical conclusions: In some situations, it should be replaced by alternative methods; in others, the closed bite registration may be the one in need of change (e.g., in surgical planning). A major challenge will be keeping protocols as simple as possible, since our goal is to provide an everyday, accessible solution.

Interestingly, the open bite protocol’s reproducibility in producing ‘*the wrong bite position exactly the same way every time*’ (as discussed earlier) may, beyond its humour value, have diagnostic potential. The clinical side of the authors – accustomed to managing CO-CR discrepancies, functional shifts, and premature contacts during complex orthodontic assessments – was quick to view the phenomenon from a diagnostic standpoint. For example, in Case II, what caused such a lateral shift in a slightly guided mandibular posture compared with free motion? Could this have diagnostic value? Such observations raise inconvenient questions: Should one ignore such findings observed during painstaking 3D modelling hard labour? If not, how does one begin the awkward conversation with an academic supervisor regarding that supervisor’s TMJ status? These unexpected observations may merit separate investigation outside the context of the main protocol. However, as with all other consistently identified trends (including those supporting previous in vitro findings), confirmation of such observations in larger cohorts will be necessary to establish their general applicability, particularly after resolution of the remaining technological constraints.

The 0.1 mm clinical tolerance level is defined by prosthodontics; in orthognathic surgery, a slightly lower accuracy standard may be acceptable. Given our authors’ background, the original aim was to develop an alternative articulation method for virtual surgery planning. That aim may be close to, if not already, achieved – but further control cases are necessary to confirm it. It may be more realistic to consider our method as a potential alternative when we examine the current evidence supporting commercially available surgical planning or aligner therapy software. In many cases, no clear protocols are described for simulating mandibular autorotation; the common response is: “*The surgeon can place the axis wherever they like, based on clinical experience*.” Such an approach will appear absurd to researchers striving for evidence-based methods. Implementing a protocol such as that proposed by Stamm et al. [8] would require minimal development effort from these companies. Neglecting basic evidence-based principles poses patient safety risks comparable to those of bite alignment accuracy errors discussed previously (S4 Text of [3] https://doi.org/10.1371/journal.pone.0329021.s008). With the methodologies presented here, we introduce a renewed digital version of the conventional analogue control setup of articulation methods based on dynamic motion registers. This approach offers a potential strategy to establish and maintain patient safety standards in clinical practice. While the integration of different digital platforms entails considerable technological challenges, the use of automated metrics and diverse comparative setups provides opportunities that clearly surpass those of the analogue experimental era. One of the main considerations is the methodological strictness of our assessment criteria. We used the least forgiving metrics to evaluate clinical applicability (*max error vs rms error* with 0.1 mm error threshold). Conventional facebow-based articulation methods met only 20–98% of comparable analogue criteria [4,13–17], and these methods had far less sophisticated means of assessing occlusal errors than our rms and maximum error metrics. Analogue facebow based articulation methods were used for nearly a century with relative success, yet our investigation defines a much higher acceptable accuracy than the analogue counterparts, while some alternatives apply minimal or no error evaluation at all [18,19].

Most of the proposed alternatives are based on anatomical information, as they evolved from traditional facebow registration techniques, now actualized to operate on virtual landmarks acquired from – *most often three-dimensional* – radiological imaging, usually using predefined prescriptions or digital counterparts of the formerly used facebow-articulator units [1,8,18,19]. Other techniques have been developed to work with radiation-free alternatives for capturing individual anatomy, such as facial scans or 3D photography. In some cases, these methods rely on prefabricated transfer keys, scan body systems, or other transfer elements to bridge the information gap between digitized facial soft tissue surfaces and intraoral scans of the maxillary arch [20–24]. Nevertheless, most of these methods (whether bone- or soft-tissue-based) assume – *similarly to traditional theory* – that static locative anatomical information can be used to identify an adequate axis of rotation (*among other key movements*) that sufficiently mimics jaw motion. A major liability emerges when the historical background is reviewed: as discussed above, analogue facebow-based articulation methods met relevant accuracy standards in only 20–98% of cases [4,12–16]. Despite this limitation, digital counterparts are usually – *if not exclusively* – validated against analogue articulation techniques, which are themselves inherently compromised. Therefore, new techniques should be validated using dynamic, individual data acquired by motion registration systems, similarly to conventional validation studies [4,12–16]. We propose a robust methodological framework for such validation, which, when combined with our earlier theoretical findings on relevant complex motion systems, may provide an adequate academic toolset for this purpose [3].

Our registration strategy focuses on a more individualized approach to acquiring rotation axis data, free of radiation, which – when combined with methods addressing other key elements of articulation (*such as mandibular protrusion and lateral excursion positions*) – may serve as suitable input for virtual articulators [25]. While the fabrication of bite plates requires minimal chairside work, most alternative techniques relying on transfer elements or scan bodies demand comparable material and time investments from clinicians.

The superiority of dynamic motion capture methods, such as optical tracking or digital axiography systems, is beyond question, as they provide comprehensive diagnostic information on TMJ function. Their primary disadvantage lies in the substantial financial and time investment required, which is often unjustified in simpler clinical scenarios. However, a recently proposed economic alternative may hold significant potential as the technology matures [26].

Taking everything into account, it appears that TMJ kinematics has diverged into two distinct worlds: one equipped with highly sophisticated motion registration systems – *seemingly from 21st-century science fiction* – such as Modjaw and digital axiography; and another mired in a ‘*digital dark age*’, where the limitations of analogue methods have been discarded without adequate replacement, leaving the field open to poorly substantiated practices. New norms and patient safety protocols must be established by the scientific community, as within the shadow cast by our ‘*shiny new toys’*, many unaddressed issues have found a convenient hiding place.

## Supporting information

S1 Table Dev. Values measured with Meshmixer’s *Deviation* tool.
(XLSX)

S2 Table The primary registration data of the two protocols: *Szeged-method* and Modjaw by the primary outputs of the algorithm and the coordinates of the acquired axes. (XLSX)

S3 Table The results of *Setup-1* Szeged. Derived parameters start at Row No. 4323.
(XLSX)

S4 Table The results of *Setup-1* Modjaw. Derived parameters start at Row No. 483.
(XLSX)

S5 Table The results of *Setup-2.* Derived parameters start at Row No. 4323.
(XLSX)

S6 Table The results of *Setup-3.* Derived parameters start at Row No. 123.
(XLSX)

S1 Video An example of a Modjaw registration sequence. (MP4)

S2 Video Visual representation of the registered axes (both *Szeged-axes* and *Modjaw-axes*) of Case I in Blender. (MP4)

S3 Video Visual representation of the registered axes (both *Szeged-axes* and *Modjaw-axes*) of Case II in Blender. (MP4)

S4 Video Visual representation of the registered axes (both *Szeged-axes* and *Modjaw-axes*) of Case III in Blender. (MP4)

S5 Video Visual representation of the registered axes (both *Szeged-axes* and *Modjaw-axes*) of Case IV in Blender. (MP4)

S6 Video Visual representation of an example of the *Setup-3* output meshes (bright) of Case I, rotated by the *Modjaw-axes*, showed in Meshmixer, compared to the ‘target’ *open* scan of the *Szeged-method* (dark). (MP4)

S7 Video Visual representation of an example of the *Setup-3* output meshes (bright) of Case II, rotated by the *Modjaw-axes*, showed in Meshmixer, compared to the ‘target’ *open* scan of the *Szeged-method* (dark). (MP4)

S8 Video Visual representation of an example of the *Setup-3* output meshes (bright) of Case III, rotated by the *Modjaw-axes*, showed in Meshmixer, compared to the ‘target’ *open* scan of the *Szeged-method* (dark). (MP4)

S9 Video Visual representation of an example of the *Setup-3* output meshes (bright) of Case IV, rotated by the *Modjaw-axes*, showed in Meshmixer, compared to the ‘target’ *open* scan of the *Szeged-method* (dark). (MP4)

S1 Presentation Detailed presentation of the *dual-bite* protocol through a case example. (PDF)
