# Supplementary material for: Status report on novel intraoral scanner-based registration method of axes of rotation of the mandible: Proof of potential applicability and technological glass ceiling of in-house development
Source: PLoS One. 2026 Feb 23;21(2):e0342893. doi: 10.1371/journal.pone.0342893 (PMC12928589; doi:10.1371/journal.pone.0342893)

STUDY CASE

Study Case of the Clinical Workflow for Dual Bite-Scan–Based Axis Registration
(*Szeged-Method*)

Associated Publication: Status report on novel intraoral scanner–based registration method of axes of rotation of the mandible: Proof of potential applicability and technological glass ceiling of in-house development

Preparation of composite plates of predefined width

(e.g., template fabricated from silicone impression material)

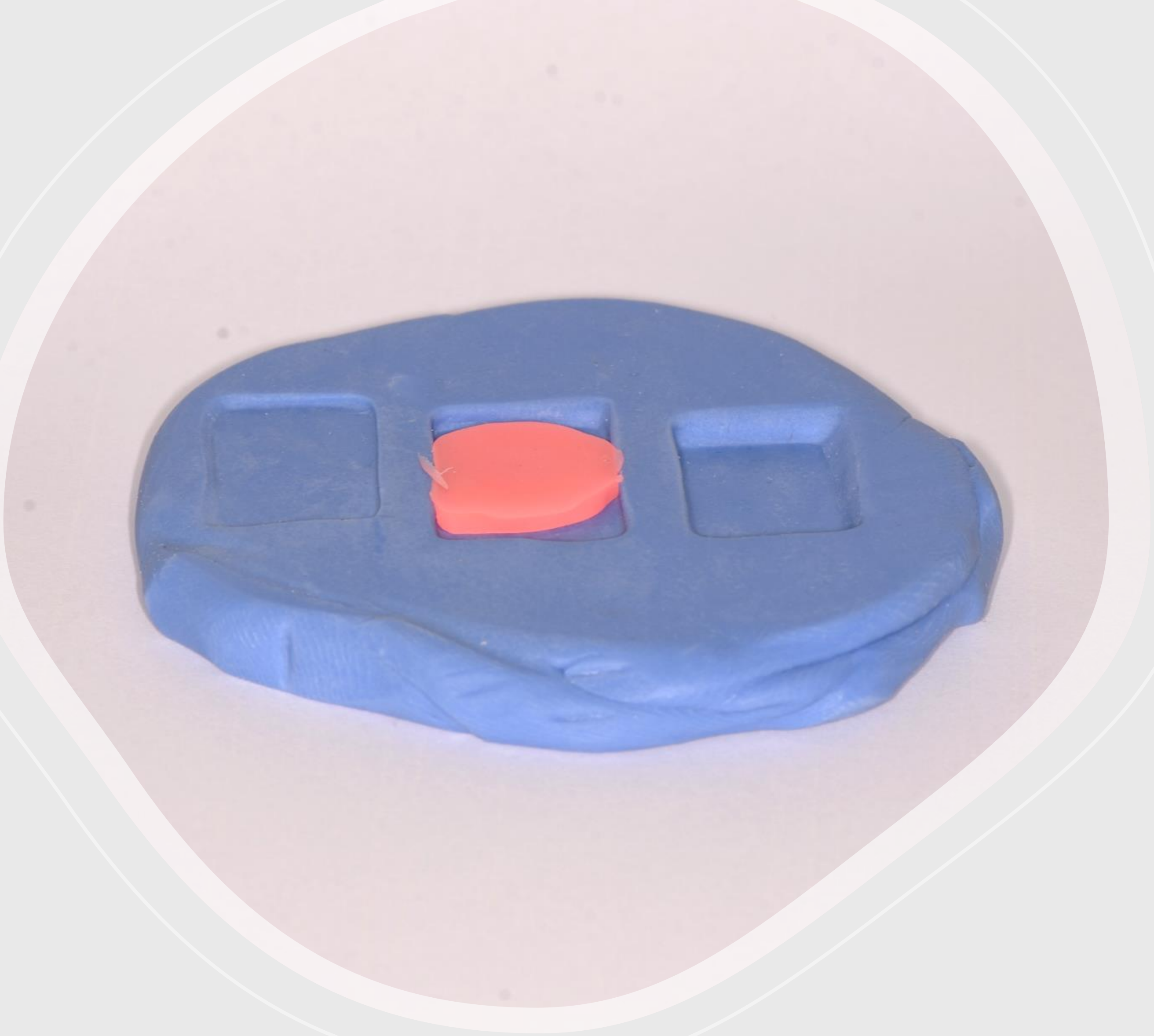

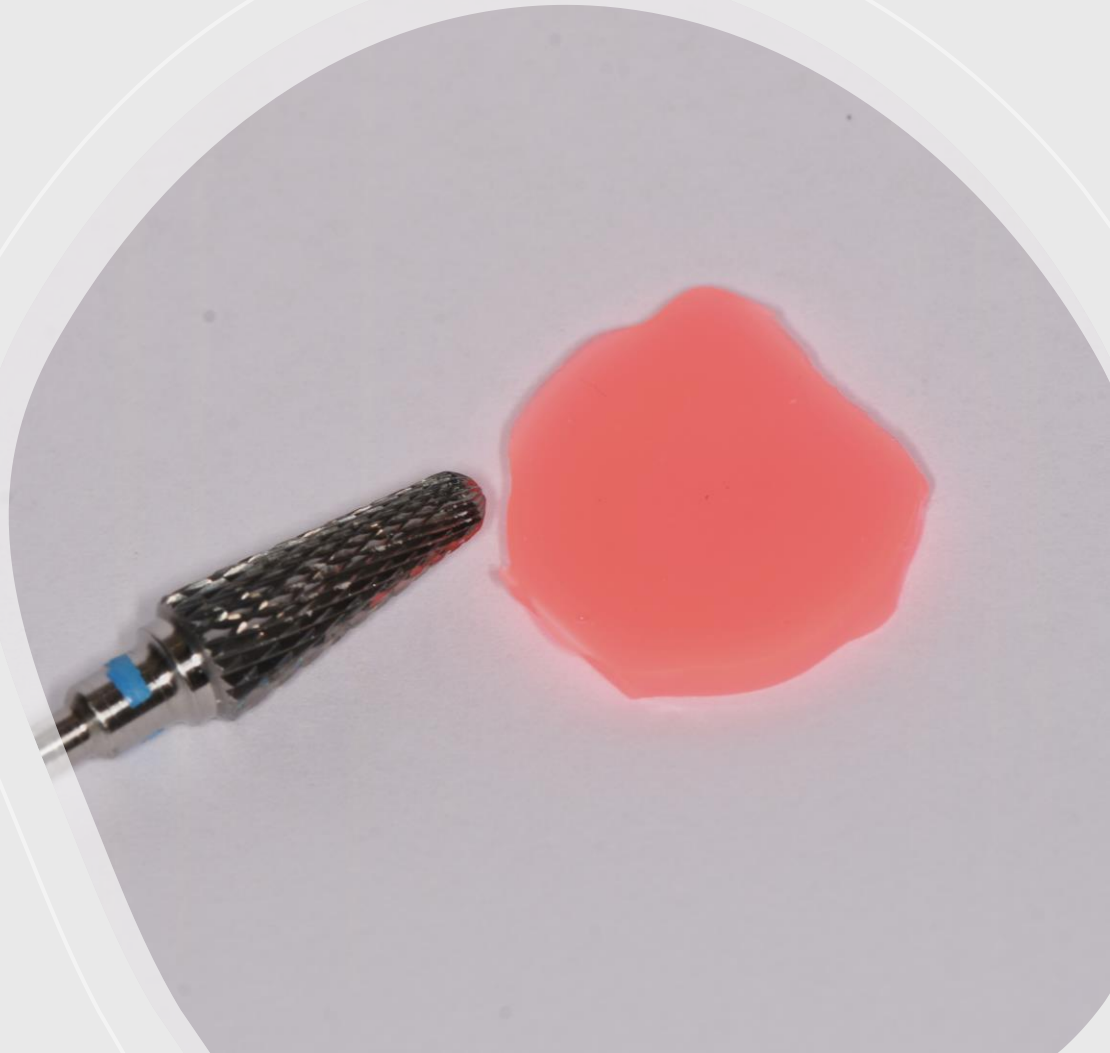

Note: For demonstrative purposes and improved visualization, coloured materials were used in this study case.

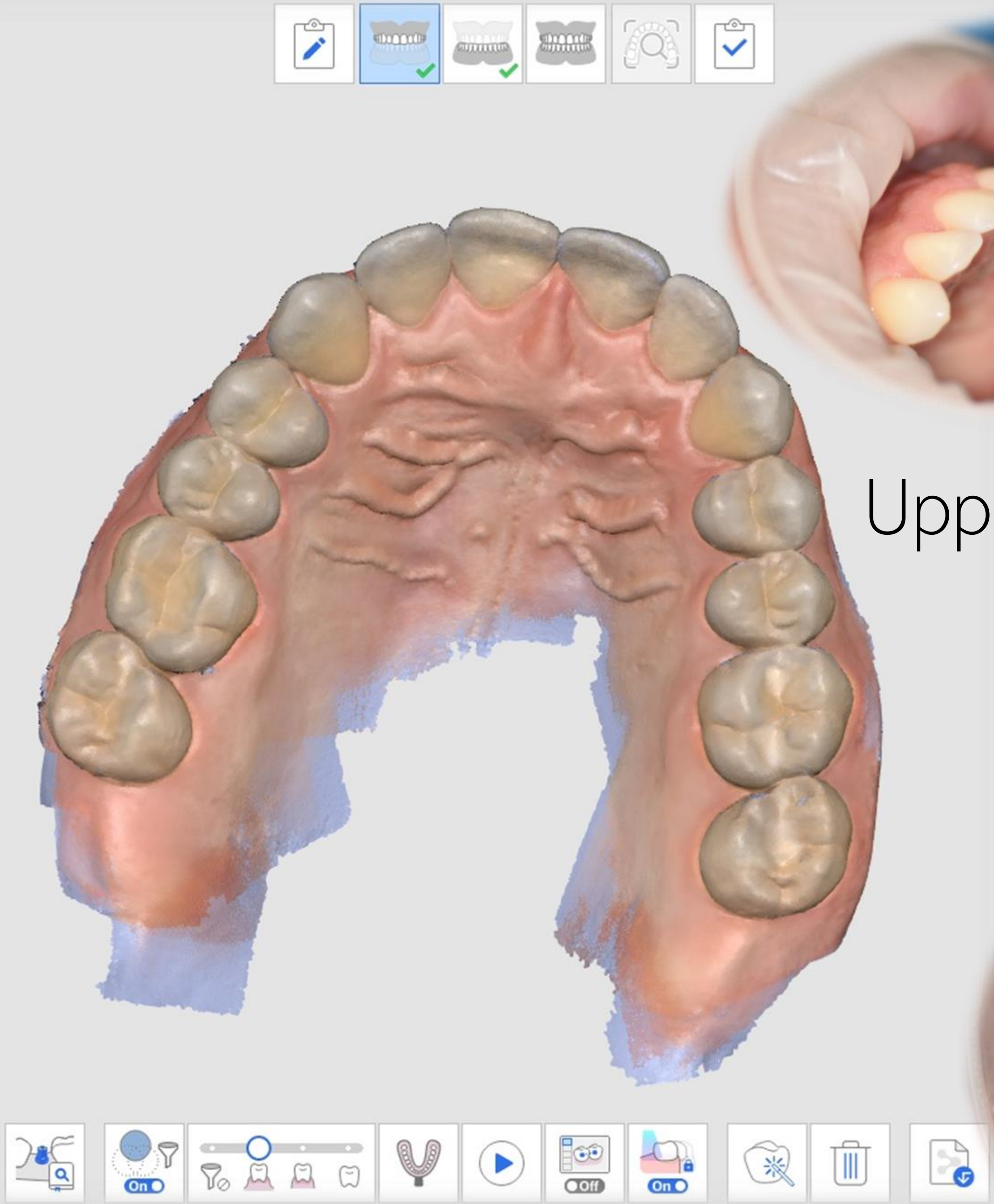

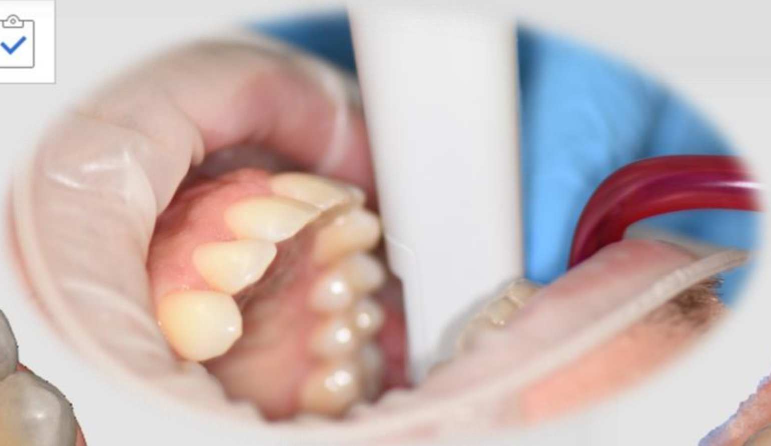

Upper & lower scan

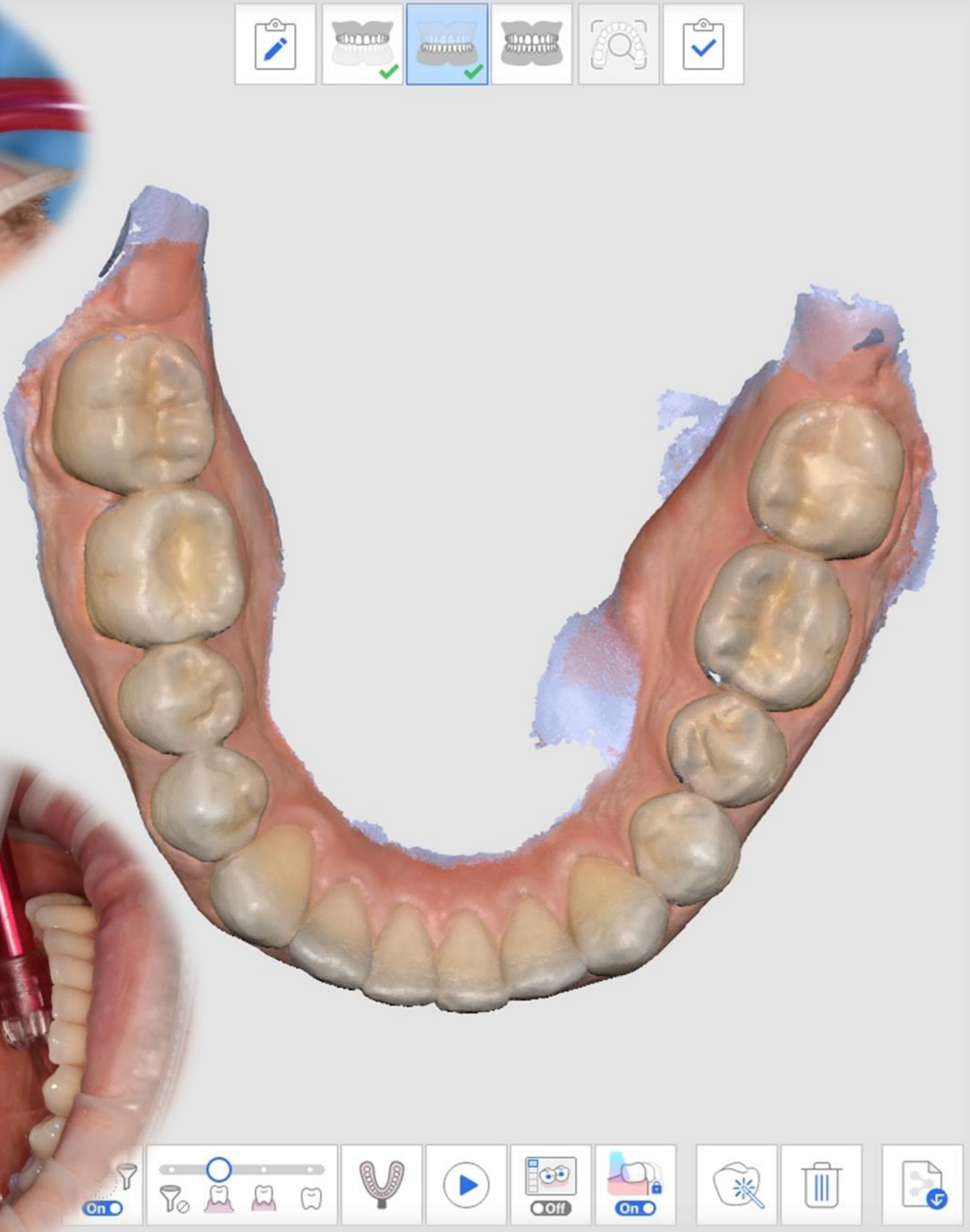

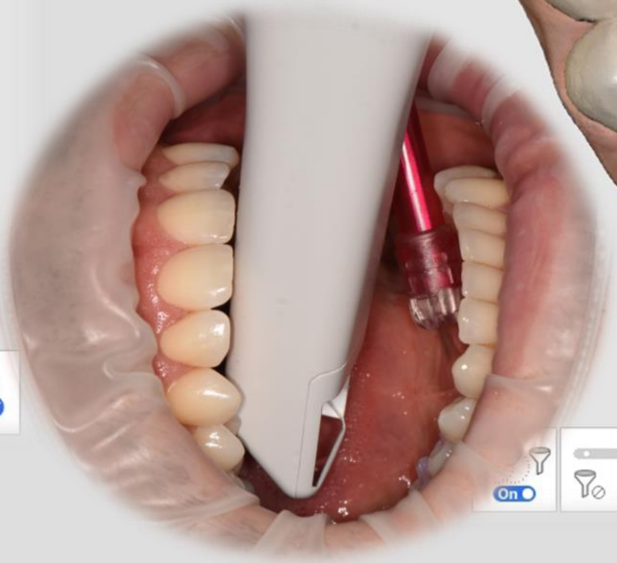

MEDIT Scan for Clinics 1.13.7

Demo Patient's Case

Simple UI

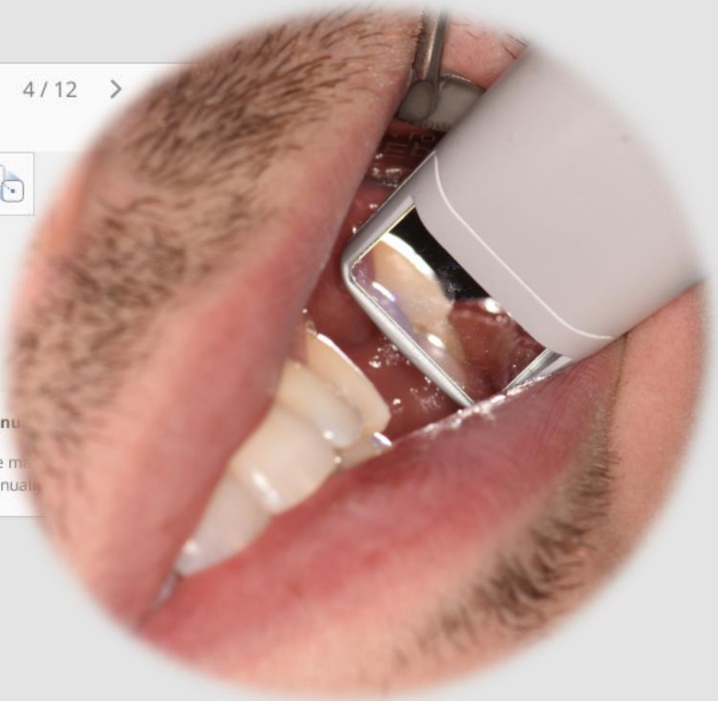

<u>Alternative strategy</u>:
Define first contact in CR if ICP≠CR

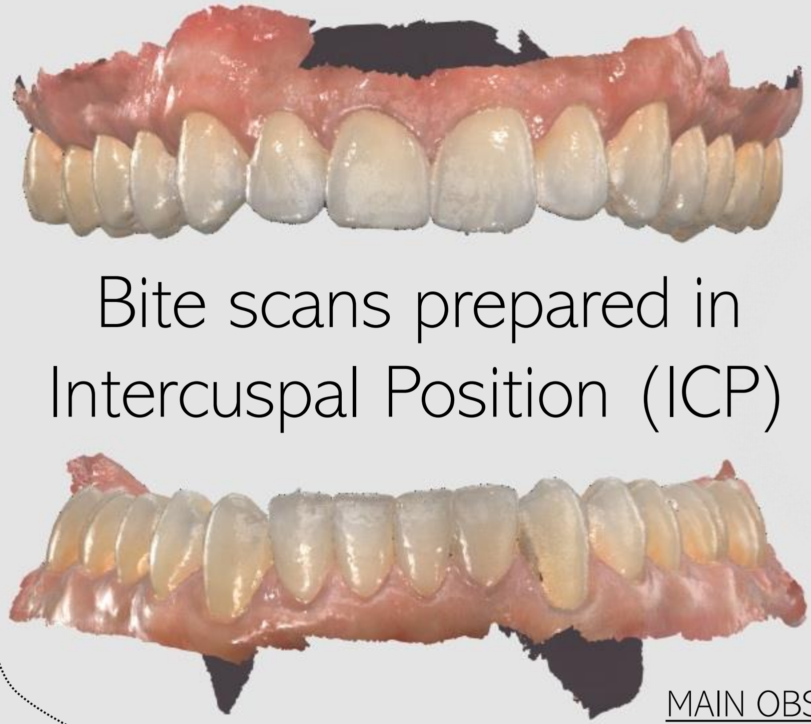

Bite scans prepared in Intercuspal Position (ICP)

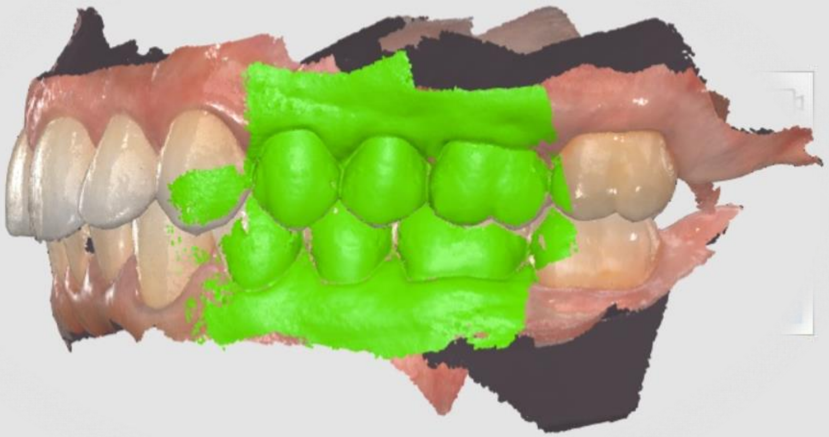

Data List

CA1

CA2

CA3

<u>MAIN OBSTACLE OF IN-HOUSE DEVELOPMENT AND RESEARCH</u>

Limited number of bite positions:

The software allows only limited cases to be captured. Ideally, an unlimited number would be needed at this stage, or the meshes would need to be post-processed before bite registration. However, because post-processing occurs only later in the workflow, surface quality becomes compromised.

All mesh surfaces must be identical and referenced to a ‚*fixed*' upper arch.

Undo Redo

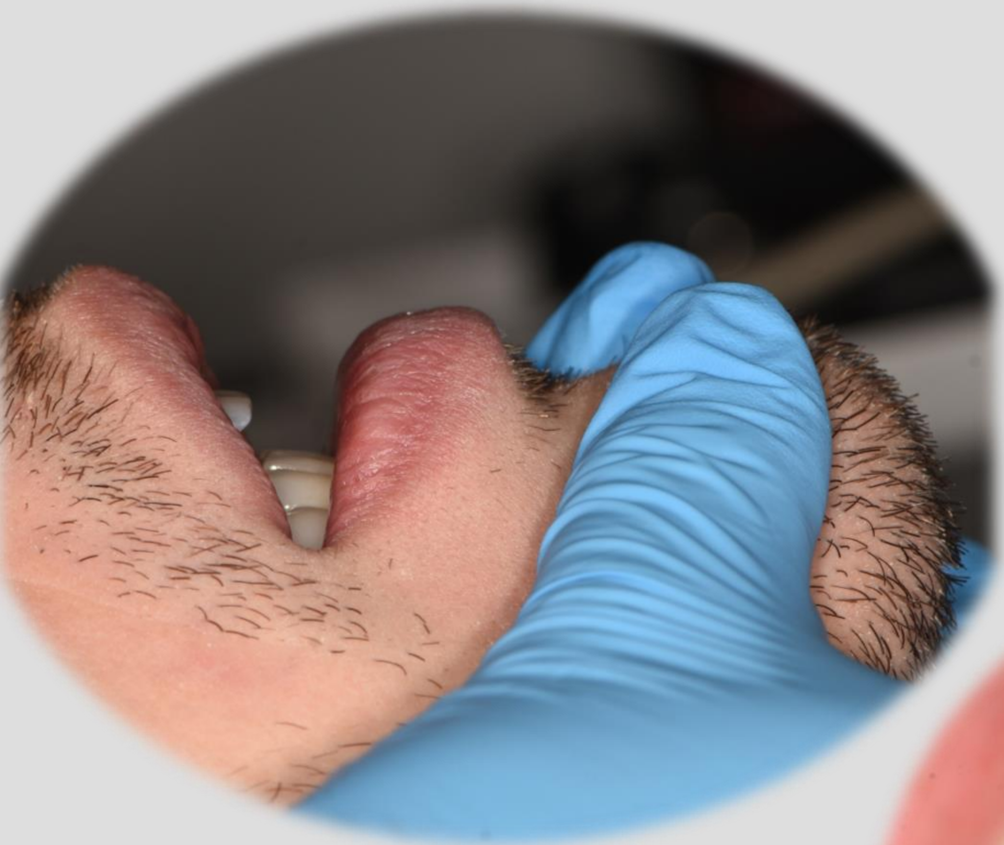

DEFINE MANDIBULAR POSITION BY GENTLE GUIDIANCE

<u>Repeated evaluation and patient education</u>:

- Relaxation
- Feedback on the first point of contact
- Repeated practice of the sensation

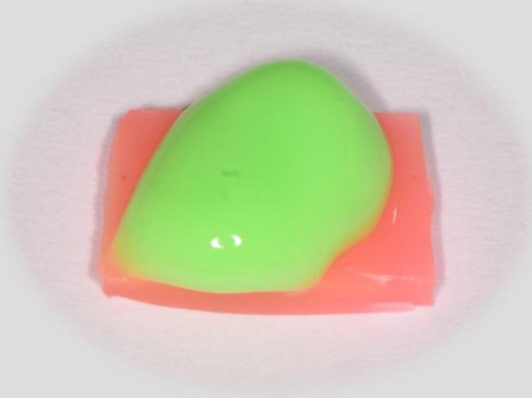

FIX BITE PLATES ON THE UPPER PRAEMOLAR REGION.

Usually composite paste material, light-cured without adhesive.

Identify first contact

Reevaluate repeatedly

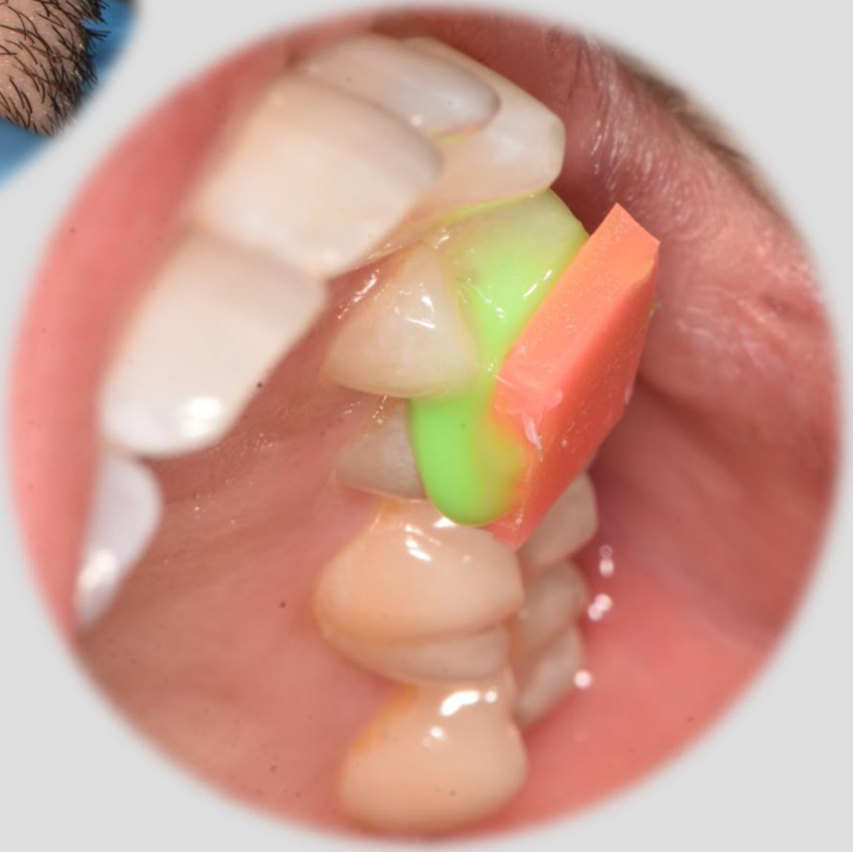

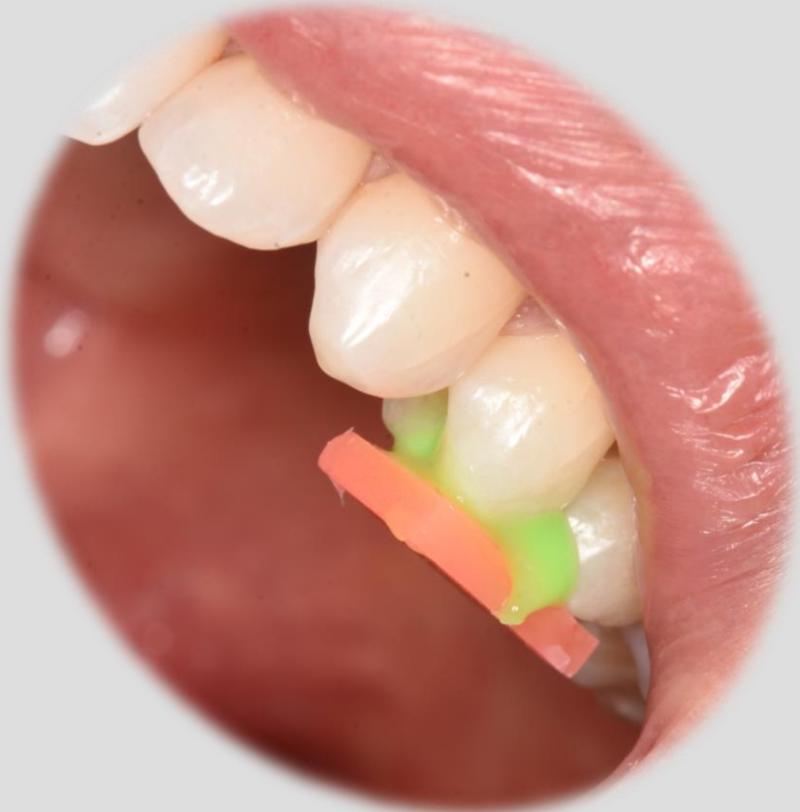

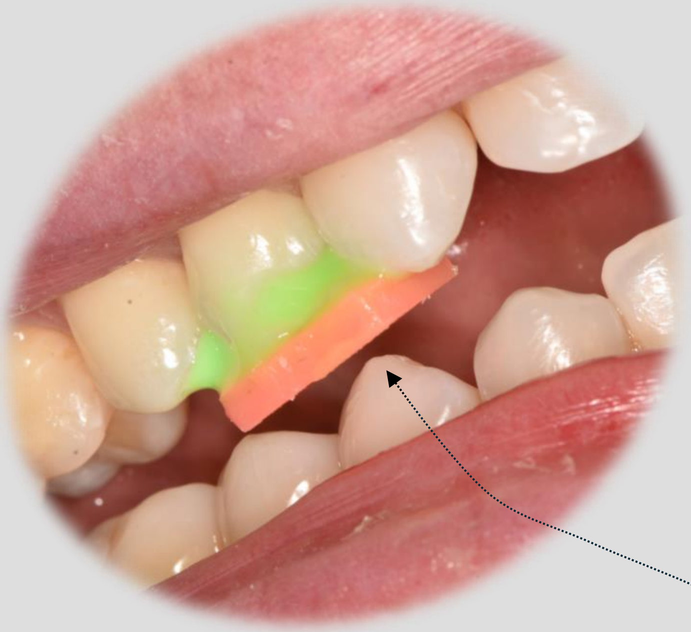

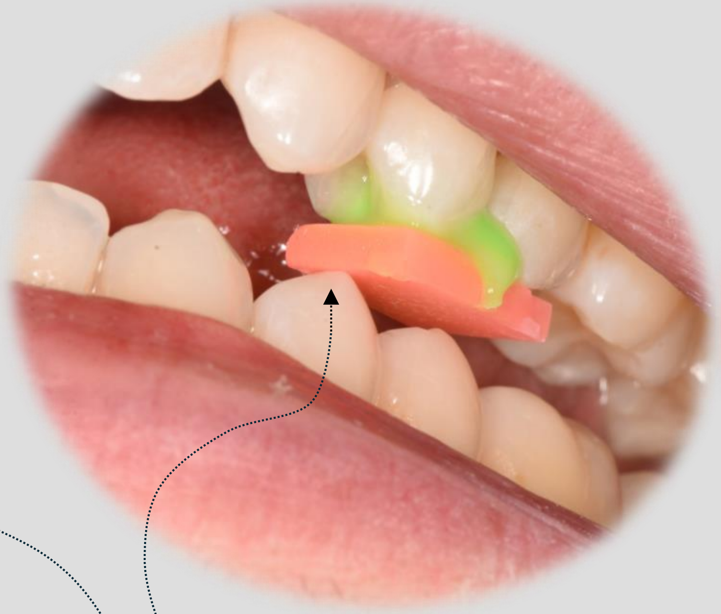

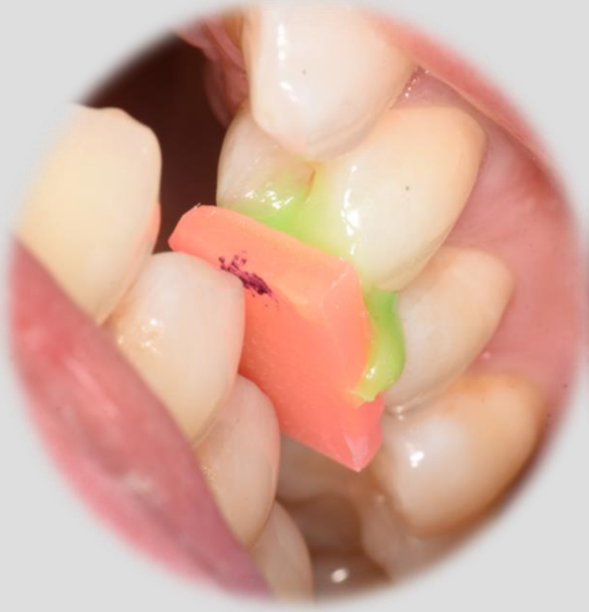

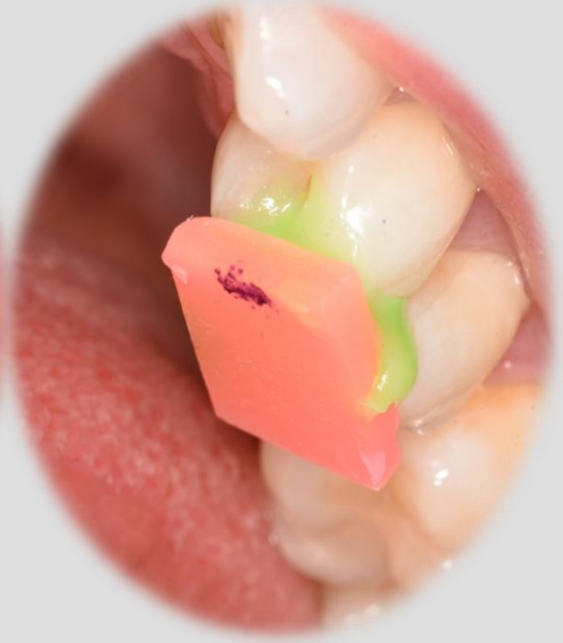

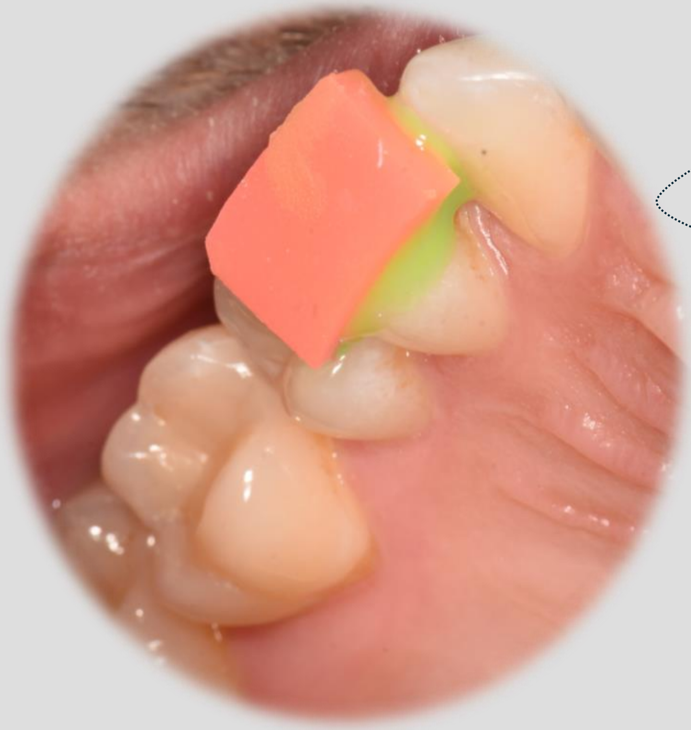

When gentle guidance does not produce bilateral simultaneous contact in the defined mandibular position:

Adjust the bite plates: add or remove material until bilateral simultaneous contact is achieved

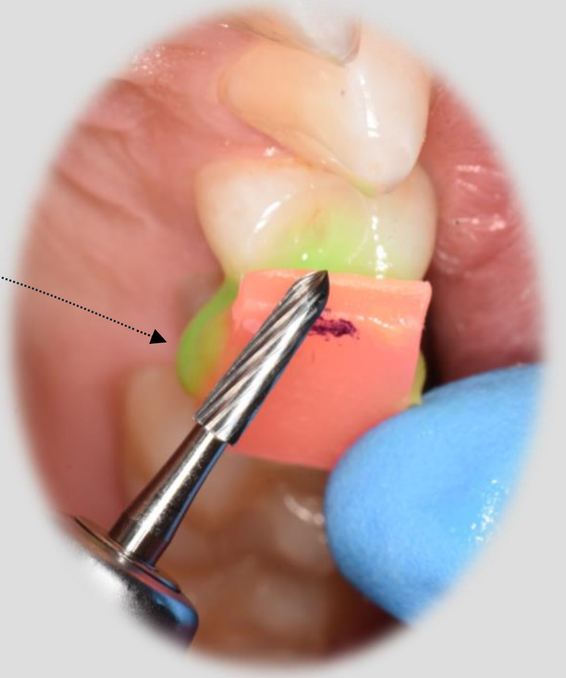

Excessive pressure during guidance must be avoided
Example: Registration for surgical planning should mimic the surgeon's manual mandibular guidance during maxillary osteotomy

Further development of different clinical strategies is necessary
(e.g., registration without guidance, deprogramming, etc.; see *Setup-3*)

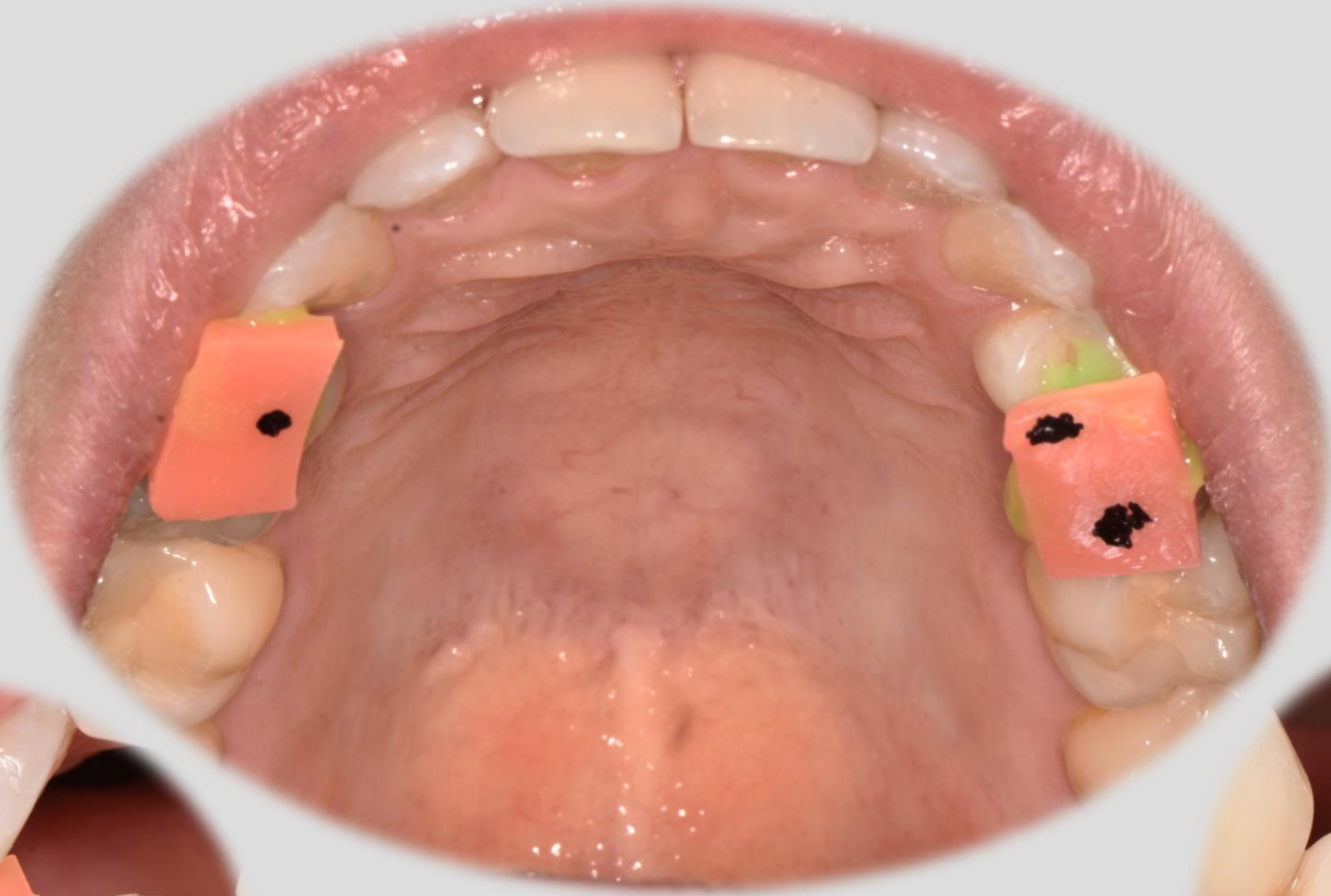

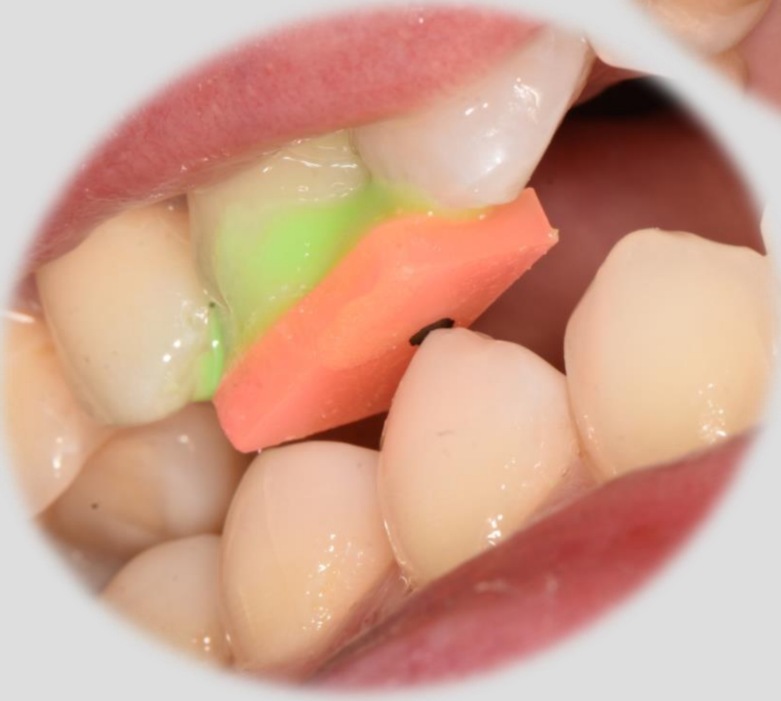

Confirm symmetry multiple times
(also via patient feedback)

Adjust bite plates until the patient consistently reports identical bilateral contact across multiple trials, with the mandible positioned as desired

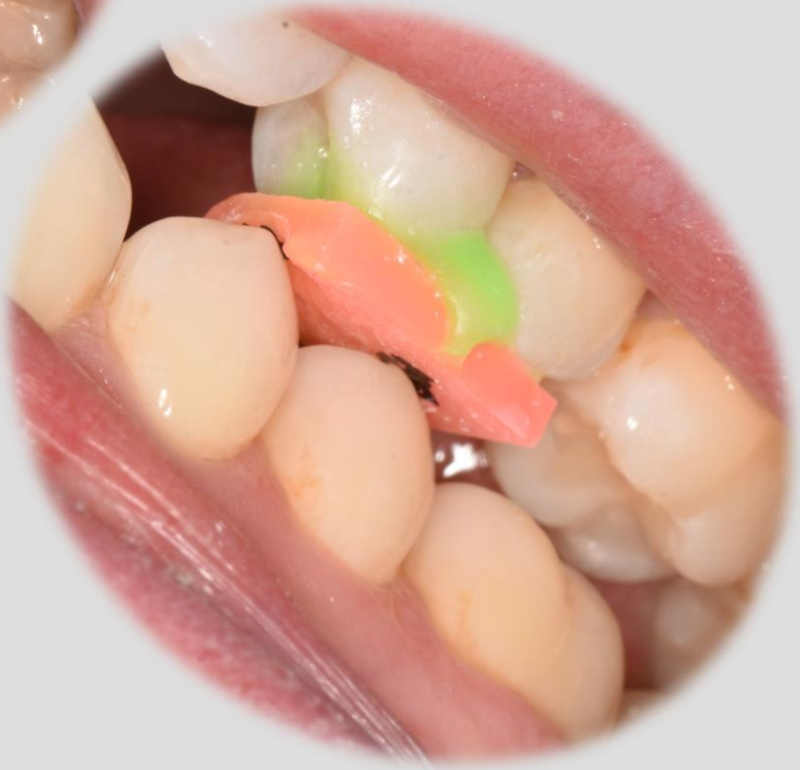

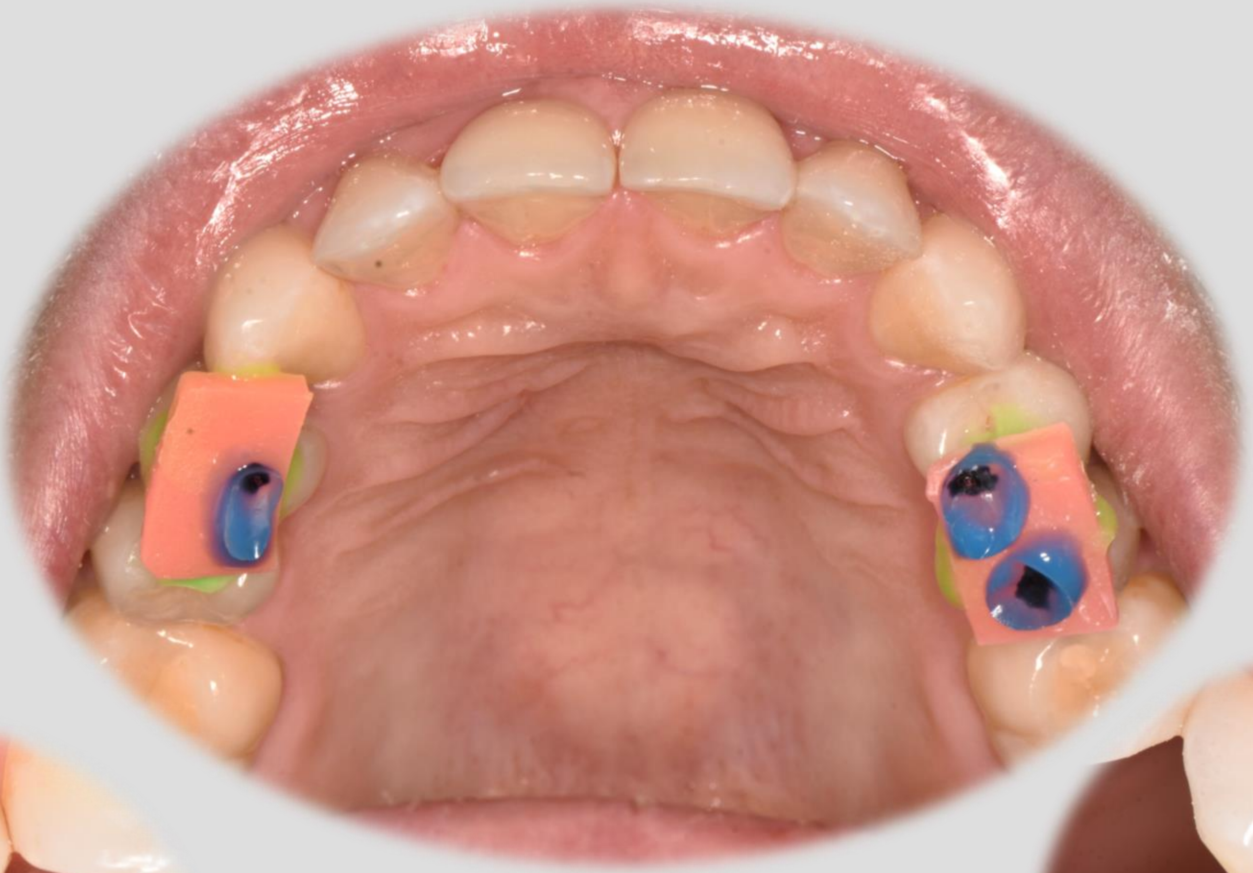

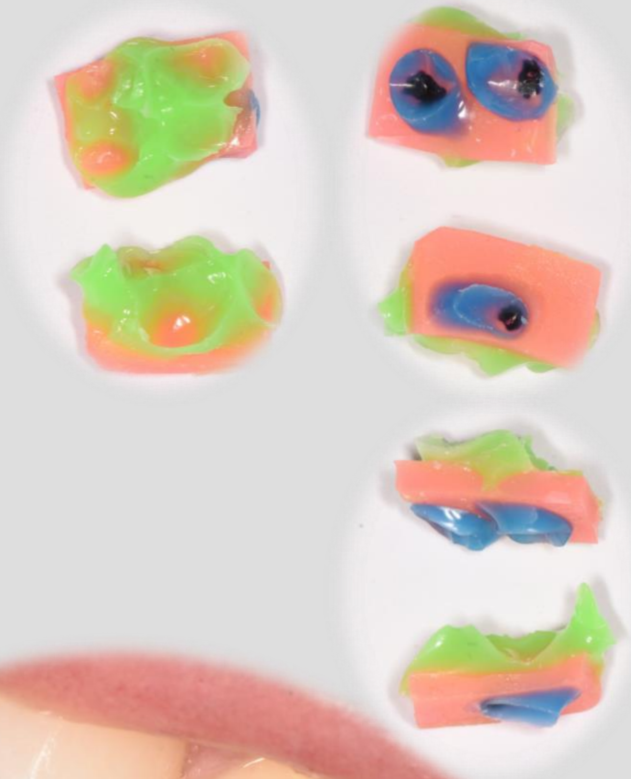

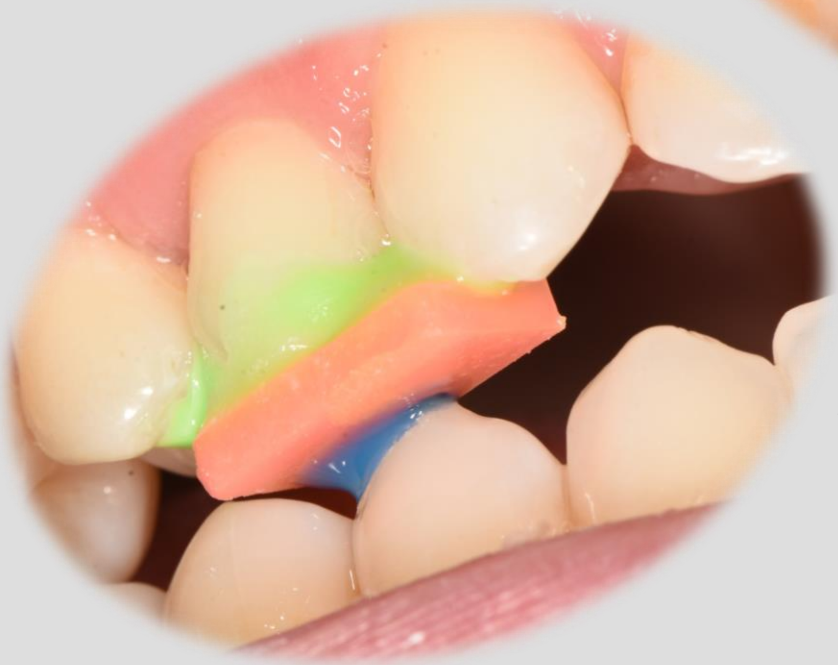

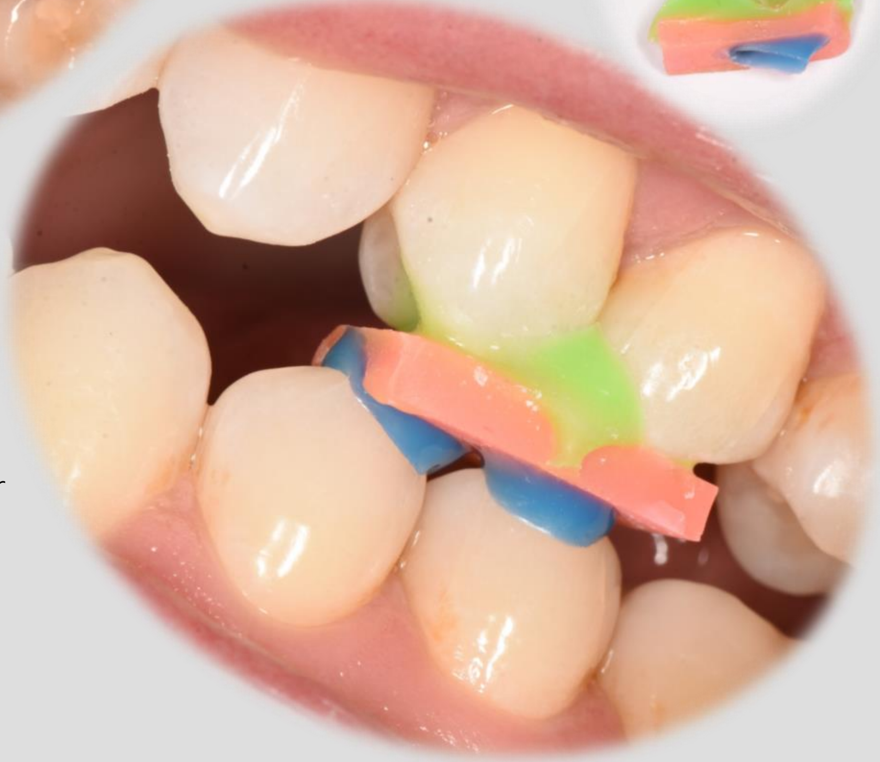

Fixation of the Achieved Position

Stabilize the established mandibular position using a flowable material.
The new material **must not alter** the defined mandibular position.

<u>Confirm multiple times:</u>

- Clear, unobstructed closure pathway
- No interferences

Bite scans performed with bite plates

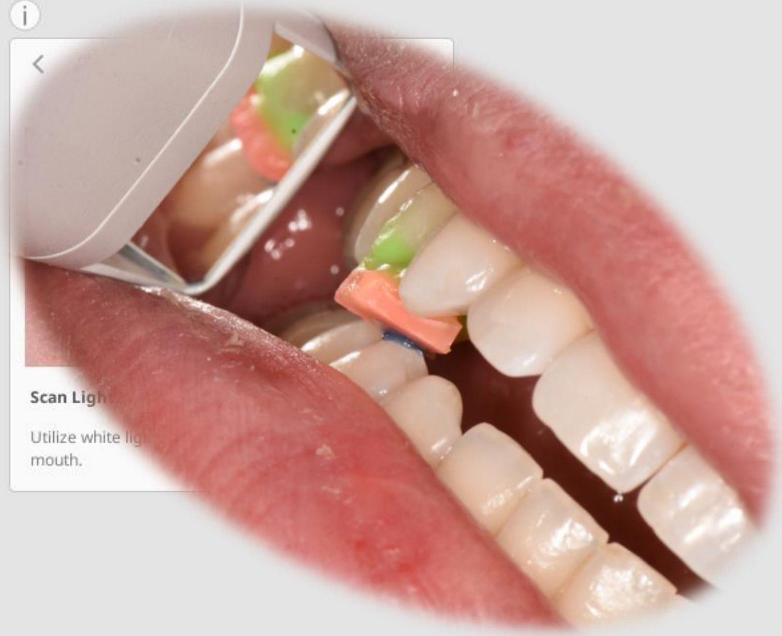

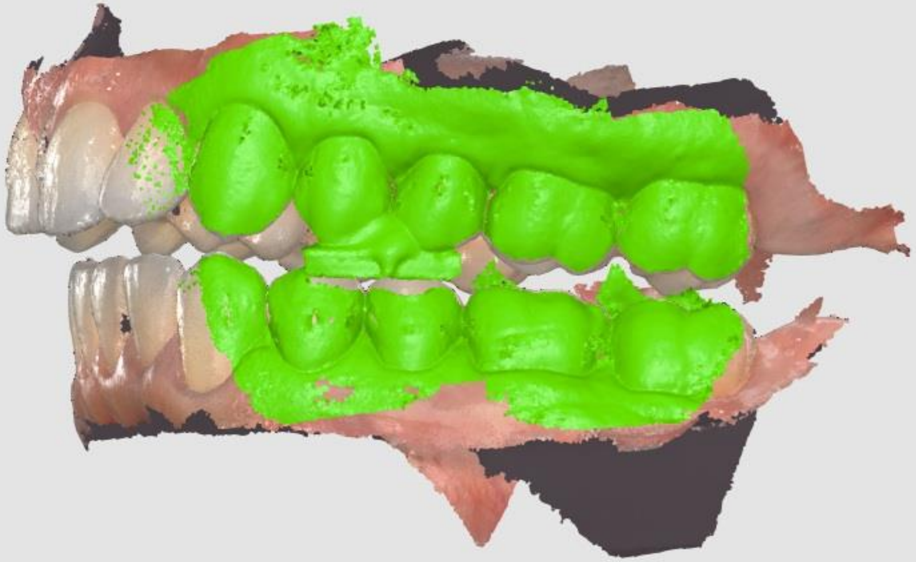

Supplement: S1 Presentation — (PDF) [file pone.0342893.s016.pdf]
